# Synergistic ANN-GA-CFD framework for high-performance Savonius wind turbine optimization with experimental validation

**DOI:** 10.1038/s41598-026-52882-0

**Published:** 2026-05-20

**Authors:** Hamdy M. Sehsah, I. M. Sakr, Ali M. Abdelsalam, Ahmed S. Saad

**Affiliations:** 1 Mechanical Power Engineering Department, Faculty of Engineering, Menoufia University, Shibin al Kawm, Menoufia 32511 Egypt; 2 Faculty of Engineering, New Mansoura University, New Mansoura, Egypt; 3 Faculty of Technological Industry and Energy, Delta Technology University (DTU), Quwaysna, 32631 Egypt

**Keywords:** Straight and twisted Savonius wind turbines, Artificial neural networks, Genetic algorithm, Computational fluid dynamics, Monte Carlo-based global sensitivity analysis, Energy science and technology, Engineering, Mathematics and computing

## Abstract

Savonius wind turbine (SWT) optimization via machine learning and optimization techniques has attracted increasing attention; however, most existing studies rely on limited datasets that cover only specific geometric parameters or operating conditions. This limitation constrains the comprehensive exploration of the Savonius wind turbine design space. Therefore, the present study constructs a comprehensive multisource dataset covering the key geometric parameters and operating conditions of SWT. Accordingly, an iterative optimization framework integrating artificial neural networks (ANN), genetic algorithms (GA), and computational fluid dynamics (CFD) is developed. The performed CFD simulations are employed to enrich the dataset by filling critical data gaps. Consequently, two high-accuracy ANN surrogate models are established for straight and twisted SWTs, achieving correlation coefficients of up to 0.98. Accordingly, optimizing the developed models results in optimal designs with maximum power coefficients of 0.1856 and 0.1927 for straight and twisted SWTs, respectively. Employing the developed ANN models with Monte Carlo-based sensitivity analysis enables the quantification of influence percentage of each design parameter and operating condition on SWT performance. Furthermore, the optimal designs are fabricated and experimentally tested under different operating conditions. The experimental measurements show good agreement with the ANN model predictions, ensuring the accuracy of the developed ANN-GA-CFD framework.

## Introduction

The increasing global demand for energy and the urgent need to reduce carbon emissions are causing a significant shift toward renewable energy sources. By the end of 2024, the total installed renewable capacity recorded 46% of the total global installed power capacity. The total installed renewable capacity during 2024 was 585 GW. Most of this increase was due to wind energy, which accounted for 19.3% of renewable energy added^1^. Wind energy is widely available and cost effective. Among the different types of wind turbines, the Savonius Wind Turbine (SWT) has gained attention because of its simple construction, low initial cost, quiet operation, acceptance of wind from any direction, and minimal maintenance requirements, making it particularly suitable for urban environments. However, its efficiency remains relatively low compared with that of other types of wind turbines^2,3^.

**Fig. 1:**
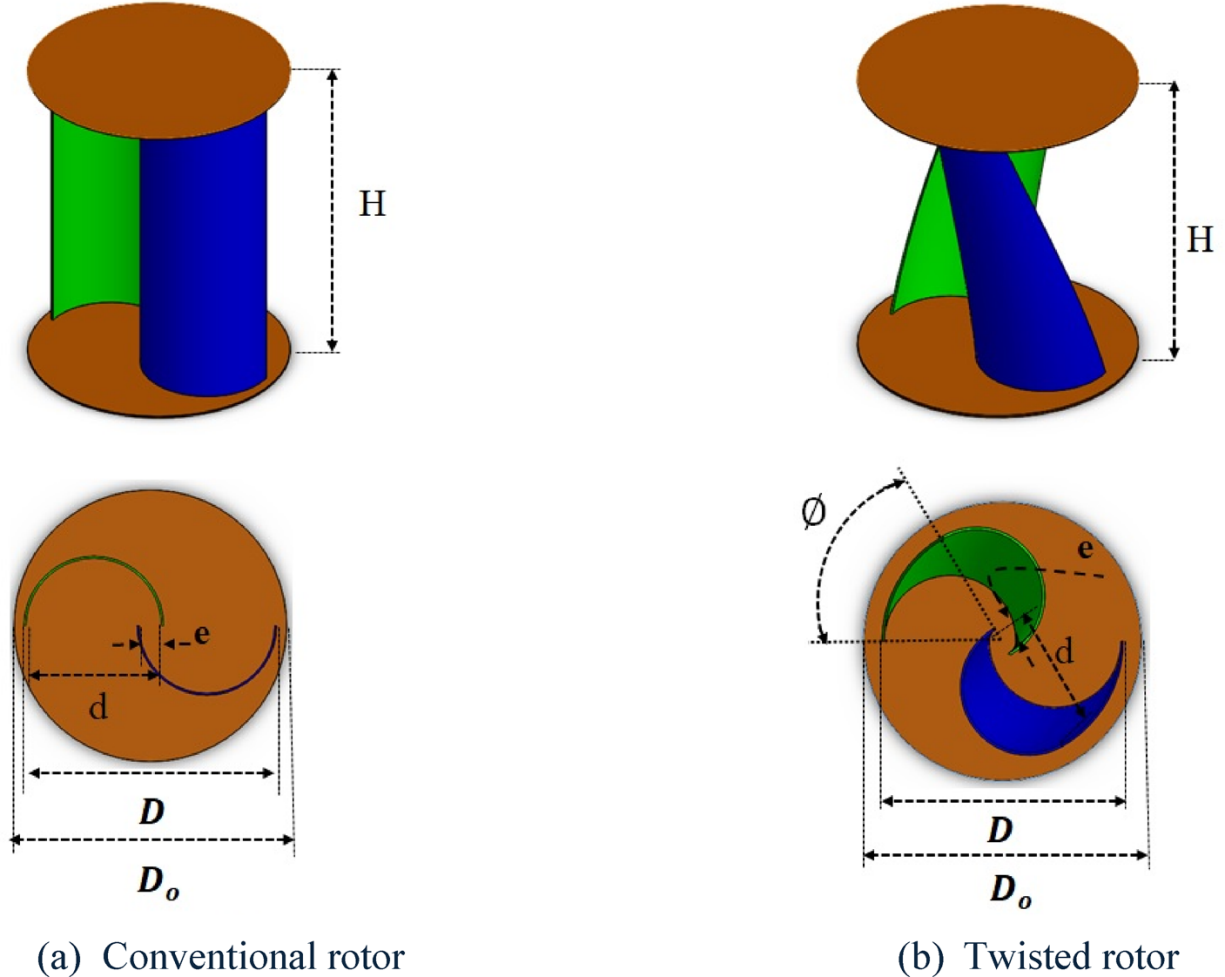
Geometric specifications of the Savonius wind turbine (SWT).

Several previous studies were performed to improve the SWT power coefficient via rotor geometric parameters or by using augmentation devices. Augmentation devices significantly increase the power coefficient, but they increase the design complexity, as the turbine performance is affected by the wind direction. This makes augmentation devices uncommon commercially. Choosing the optimum geometric parameters and operating conditions leads to better performance^4^. Figure 1 illustrates the SWT configuration, highlighting the principal geometric design parameters that define its aerodynamic and structural characteristics, as reported in^5^. These parameters include:

1 $${\mathrm{Aspect}}\;{\mathrm{ratio}}\;\left( {{\mathrm{AR}}} \right) = \frac{H}{D}$$

2 $${\mathrm{Overlapratio}}\;\left( {{\mathrm{OR}}} \right) = \frac{e}{d}$$

3 $${\mathrm{Endplateratio}}\;\left( {{\mathrm{EPR}}} \right) = \frac{{D_{o} }}{D}$$

4 $${\text{Tip speed ratio}}\left( \lambda \right) = \frac{{{\boldsymbol{u}}_{{\boldsymbol{t}}} }}{{\boldsymbol{V}}}$$

5 $${\mathrm{Reynolds}}\;{\mathrm{number}}\;\left( {{\mathrm{Re}}} \right) = \frac{\rho VD}{\mu }$$

6 $${\mathrm{Power}}\;{\mathrm{coefficient}}\;\left( {{\mathrm{C}}_{{\mathrm{p}}} } \right) = \frac{{P_{rotor} }}{{P_{available} }} = \frac{\Omega T }{{0.5 \rho A V^{3} }}$$

where $H$ is the rotor height, $D$ is the rotor diameter, $e$ is the overlap distance, $d$ is the bucket diameter, ${D}_{o}$ is the end plate diameter, $\rho$ is the air density, $\mu$ is the dynamic viscosity of air, $\Omega$ is the angular velocity, $T$ is the torque, $A$ is the swept area, and *V* is the wind velocity.

The following studies were performed via a traditional approach to analyze and optimize the performance of SWT at different geometric parameters and operating conditions. Kamoji^6^ experimentally studied the effects of AR, OR and Re for straight and 90° twisted two-bladed single-stage rotors with EPR = 1.1. Zhao^7^ numerically studied the effects of the number of blades ${N}_{b}$(2,3), AR (1–7), OR (0–0.5) and rotor twist angle $\emptyset$ (90–360)° for single-stage roto without end plates. The best design was achieved at (AR 6, OR 0.3, twist 180° and two bladed roto). Lee et al.^8^ numerically and experimentally studied the $\emptyset$ effect on SWT performance. The twist angles studied were 0 $^\circ$, 45 $^\circ$, 90 $^\circ$, and 135 $^\circ$. The maximum ${C}_{p,max}$ was achieved at 45°. Fujisawa^9^ performed experimental studies on the effect of OR (0–0.5) on the rotor static torque and rotor performance, and the highest ${C}_{st}$ and ${C}_{p}$ were achieved at OR = 0.15. Chen Jian^10^ studied the effect of OR (0–0.33) on one- and two-stage rotors, and the highest ${C}_{p}$ was achieved at a single-stage rotor with OR = 0.167. Jeon et al.^11^ studied the effects of end plates with various shapes and sizes on 180° twisted rotors. Notably, the use of end plates increases ${C}_{p}$ by 36%, linearly with increasing end plates.

Wang et al.^12^ experimentally and numerically investigated ${C}_{p}$ and ${C}_{t}$ for eight different end plate geometries and sizes with straight SWT. The endplates improve performance by 299.7%, as they reduce tip flow leakage, but a high EPR increases drag, which reduces ${C}_{p}$. Anbarsooz^13^ experimentally and numerically investigated the performance of SWT at $\emptyset$ (0 $^\circ$, 30 $^\circ$, 45 $^\circ$) and Re (150,000, 184,000). The best performance was achieved at twist = 0° and Re = 150,000. Damak et al.^14^ studied the performance of 90° twisted SWT at Re (73,700–131,600). The maximum power coefficient was achieved at Re = 121,000. Ramadan et al.^15^ experimentally studied two- and three-bladed SWT at Re (110,000–180,000), AR = 1, OR = 0.296 and EPR = 1.1, and the best performance was achieved for two bladed rotors at Re = 1,250,000. Simsek^16^ experimentally studied C_p_ for straight and 45° twisted SWT with ${N}_{b}.$(2, 3, 4 and 5) with AR = 1.33 and without end plates. The best performance was achieved by the three-bladed straight rotor. Kothe et al.^17^ compared 180° twisted rotors with two-stage straight rotors, both of which have two blades, OR = 0.15, Re = 46,300 and total AR = 4. However, twisted manufacturing is more complicated than two-stage rotors and results in a higher ${C}_{p}$ and more stable torque.

Optimizing the geometric design and operating conditions of SWTs is a complex and high-dimensional problem. Artificial intelligence (AI) offers powerful tools to address this challenge via data from previous studies. ANN can capture complex nonlinear relationships and have been used in previous SWT studies aiming to use the model for predicting performance in the design space represented by the model. The other objective was to use the model with an optimization technique to obtain the optimum design that achieves the highest performance. Table 1 summarizes the previous studies on SWT that have used AI modeling for prediction or optimization. Rathod et al.^18^ compared two modeling techniques, ANN and genetic expression programming (GEP). Notably, the traditional mathematical functions used by GEP cannot represent SWT complex data, and the ANN achieves a better coefficient of determination^18^.

**Table 1:** Summary of previous studies employing AI-based modeling and optimization for Savonius wind turbines.

Author	Objective	Dataset source	Input	Output	AI modeling method -optimization technique
Prediction					
Sargolzaei et al.^19^	Predicting ${C}_{p}$ and T for 6 different rotors each one has different (OR)	Experimentally generated	Rotor angle and $\lambda$	${C}_{P}$	ANN
Debnath and Das^20^	Predicting ${C}_{p}$ and ${C}_{t}$ for 3 bucket SWT rotor	Experimental generated	OR, $\lambda$, and Angular velocity	${C}_{P}$	ANN
Biswas et al.^21^	Performance evaluation of hybrid 3 bladed Darrieus 3 bladed Savonius VAWT	Experimentally generated	Wind speed, $\lambda$, and RPM	${C}_{P}$	ANN
Rathod et al.^18^	SWT performance prediction, considering several geometric parameters using 2 different modeling techniques and comparing them	Experimentally generated	Shape factor, Nb, Nst, overlap length, AR, ER, Wind tunnel cross area	${C}_{P}$	(ANN–GEP) modeling
Optimization					
Mohammadi et al.^22^	Optimizing SWT for maximizing ${C}_{p}$ using GA	Kamoji et al.^7^	AR, OR, $\emptyset$(0&90°), shaft existence, Re, and $\lambda$	${C}_{P}$	ANN–GA
Storti et al.^23^	Optimizing deflector shape and size for reducing SWT reverse Torque	2D CFD generated	6 parameters (angles & lengths) represent deflector size, shape, and position	${C}_{P}$	ANN–GA
Svorcan et al.^24^	Design an optimal flow concentrator for VAWT	3D CFD generated	4 parameters (lengths) represent its shape	V_mean_ -V_min_/ V_max_	ANN–GA
Hashem and Zhu^25^	Bioinspired Savonius-type hydrokinetic turbine optimization using evolution algorithm and perform sensitivity analysis	2D CFD generated	OR and Gap ratio	${C}_{P}$	ANN–Evolution Algorithm
Haddad et al.^26^	Optimizing SWT with additional blades + Sensitivity analysis	2D CFD generated	Blade shape factor, Blade arc angle, Additional blade radii ratio, and Additional blade angle	${C}_{P}$	ANN–GA
Torres et al.^27^	Optimizing SWT	3D CFD generated	AR, OR, and $\emptyset$	${C}_{P}$	Response surface–GA
Hosseini et al.^28^	Optimizing SWT	3D CFD generated	AR, OR, and $\emptyset$	${C}_{P}$	ANN–GA
Abdel-razak et al.^29^	Optimizing key configuration parameters of a twin two-stage SWT	3D CFD generated	Turbine spacing, Relative angle and Rotation direction	Average ${C}_{P}$	Taguchi Method
Paniagua-García et al.^30^	Redesign the cross-sectional profile of a Savonius turbine blade to maximize its ${C}_{p}$	2D CFD generated	Blade description coordinates	${C}_{P}$	ANN–GA
Kumar et al.^31^	Optimize the layout of a closely spaced array of four Savonius rotors	3D CFD generated	Positions (coordinates) of four rotors within a constrained area	${C}_{P}$	ANN–PSO

Despite the promising efficiency of artificial intelligence (AI) methods, especially artificial neural network (ANN), integrated with a genetic algorithm (GA) for modeling and optimizing the performance of the Savonius wind turbine (SWT), some limitations remain in the currently available studies. First, the majority of existing works predominantly rely on limited datasets, typically derived from a single source, either fully self-generated datasets or collected from a small number of published studies. Although such datasets simplify model construction, they often represent SWT performance only within specific geometric configurations and neglect other parameters effects, especially operating conditions. Consequently, the design space explored during the optimization process is inherently constrained. A further key limitation is the lack of experimental validation for optimized SWT designs via AI and optimization techniques, which makes their practical performance and reliability uncertain.

To address these gaps, the current study aims to develop a comprehensive multisource dataset of key SWT geometric parameters, including the aspect ratio (AR), overlap ratio (OR), end plate ratio (EPR), number of blades (N_b_), and twist angle ($\emptyset$), in addition to operating parameters, including the Reynolds number (Re) and tip speed ratio (λ). Data are systematically gathered from literature and augmented by targeted CFD simulations to fill critical gaps within the design and operating space. The resulting dataset is used to develop high-accuracy ANN models that represent straight and twisted SWT performance over wide ranges of geometric and operating parameters. This enables a robust optimization process capable of exploring a significantly expanded design and operating space. Moreover, Monte Carlo-based global sensitivity analysis is performed via the developed ANN models to quantify the relative influence of each design parameter and operating condition on SWT performance, thus offering practical guidance for future SWT design and operational control. Finally, the optimal SWT configurations are fabricated and experimentally tested under various operating conditions. The experimental results are compared with ANN predictions to validate the accuracy of the current proposed framework.

## Methodology

**Fig. 2:**
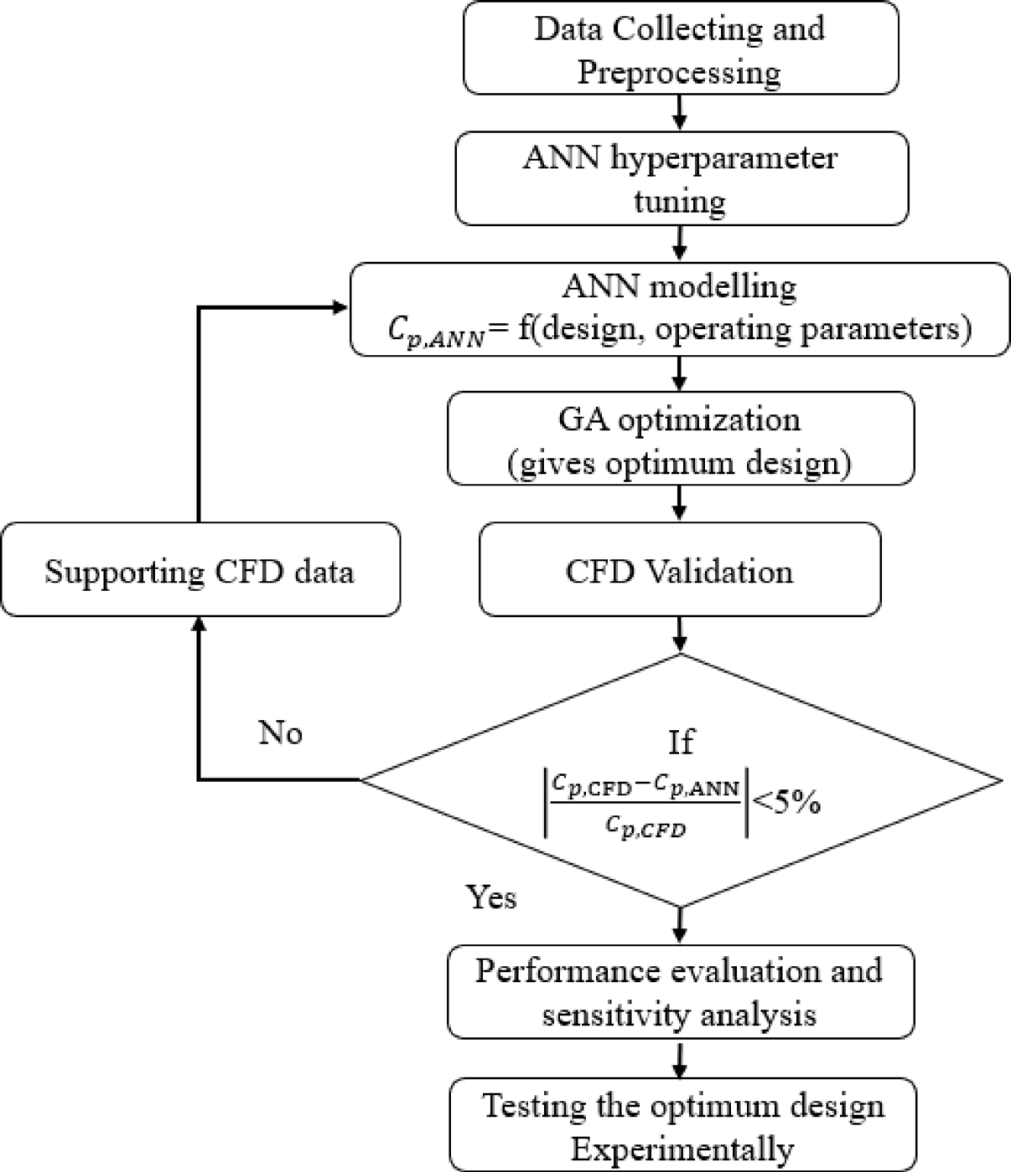
Flowchart illustrates the methodology of the proposed ANN-GA-CFD optimization framework.

This study employs an integrated ANN-GA-CFD framework, presented in the methodology flowchart shown in Fig. 2. The proposed methodology includes eight sequential steps, beginning with data collection along with preprocessing and concluding with experimental testing of the optimal turbine designs. The intermediate steps include (2) ANN hyperparameter tuning to identify a suitable ANN structure, (3) training the specified ANN structure, (4) using a GA to specify the optimal parameters that maximize performance, (5) performing CFD simulation for the optimal result to obtain ${C}_{p,CFD}$, and (6) comparing ${C}_{p,ANN}$ and ${C}_{p,CFD}$ for the optimal result. If convergence is not achieved, the generated CFD case is incorporated into the training dataset to increase model accuracy in poorly represented regions of the design space. This ANN-GA-CFD iterative loop is repeated, and the targeted augmenting CFD samples are generated through this loop until convergence is attained. (7) The converged ANN model is then used for SWT performance evaluation and sensitivity analysis.

### Data acquisition and preprocessing

The generalization ability of a data-driven surrogate model is contingent on the breadth and quality of its training data. To construct a model representative of the Savonius turbine design space, a multi fidelity dataset was collected from peer-reviewed experimental and numerical studies on semicircular-bladed SWT rotors, as represented in Table 2. The following key performance governing parameters were systematically extracted: AR, OR, EPR, ${N}_{b}$, $\emptyset$, $\lambda$, and Re, with ${C}_{p}$ as the target variable. To ensure consistency across diverse sources, all the parameters were recalculated and validated via Eqs. (1–5). The Z score filtration method was used for outer layer detection and removal^32^. The filtration was applied per variable, and the utilized Z-score threshold was 3. The filtration process has reduced the dataset from 1089 to 867 samples. In addition to all the data input parameters are then normalized in the range [0, 1] to enhance the numerical stability and training efficiency of the ANN. Table 2 presents the filtered collected data sources, range of each parameter, and maximum ${C}_{p}$ achieved in each study, in addition to the total collected data minimum, maximum and skewness for each parameter.

**Table 2:** Summary of the collected dataset used for SWT modeling and optimization.

Reference	Study	AR	OR	Re	$\emptyset$	EPR	${N}_{b}$	$\lambda$	${C}_{p, max}$
Fujisawa^9^	Experimental	1	0:0.5	145,230	0	1.1	2	0.07: 1.536	0.169
Hayashi et al.^33^	Experimental	0.88	0.277	14,000: 420,000	0	1	2	0.231: 1.38856	0.174
Kamoji et al.^34^	Experimental	1	0.15	77,600: 155,000	0	1.1	2	0.49425	0.173
Saha et al.^35^	Experimental	0.8	0	8913: 151,023	0	1.1	2 & 3	0.654: 0.815	0.174
Kamoji et al.^6^	Experimental	0.88: 1.17	0:0.3	5770: 201,958	0 & 90	1.1	2	0.08: 1.406	0.18
Anbarsooz^13^	3D CFD	1	0	150,000: 184,000	0:45	1.067	2	0.298: 1.202	0.119
Lee et al.^8^	Experimental	1.33	0.127	319,707	0: 135	1	2	0.45: 0.95	0.1268
Ramadan et al.^15^	3D CFD	1	0.296	109,260: 178,713	0	1.1	2 & 3	0.185: 1.398	0.14
El-Askary et al.^36^	Experimental	1	0.15	163,400	45	1.1	2	0.11: 1.42	0.181
Shamsuddin and Kamaruddin^37^	Experimental	0.75	0	49,000: 88,208	0	1.1	2	0.4: 0.96	0.102
Wang et al.^12^ symmetrical walls	3D CFD	1	0.295	180,000	0	1: 1.3	2	0.2:1.2	0.139
Total collected data specifications	Min	0.75	0	49,000	0	1	2	0.07	0
	Max	1.33	0.5	420,000	135	1.3	3	1.54	0.181
	Skewness	0.604	0.49	2.144	0.681	0.349	2.54	− 0.177	− 0.27

Blockage correction hasn’t been used during data preprocessing because most of these sources have used open jet wind tunnels where blockage effects are minimal due to unrestricted flow entrainment. Whereas the studies in refs.^9,33,37^ which performed inside a confined test section, have a blockage ratio below 5%. For Re similarity, rather than assuming Reynolds similarity across studies, we explicitly included Reynolds number (Re) as an input parameter to the ANN. This allows the model to learn Re-dependent Cp behavior directly from data, without imposing a prior similarity assumption. It’s worth mentioning that carefully selecting data sources and adopted preprocessing reduces inconsistency between sources but cannot completely remove residual inter-study bias.

### Artificial neural network (ANN)

The development of an ANN requires hyperparameter tuning to identify a suitable ANN structure, particularly with multisource data, to avoid the risks of overfitting or underfitting^38^. MATLAB R2023b was used for ANN hyperparameter tuning and modeling. The dataset was randomly divided into 70%, 15%, and 15% for training, validation, and testing, respectively. The hyperparameter tuning process was performed only on the 70% training subset. The external 15% validation set was reserved for independent verification of the selected model. The 15% test set was strictly held out and used only once for the final evaluation of model performance, ensuring an unbiased assessment of generalization capability. Grid search was employed for systematically traying all specified hyperparameter configurations while considering the number of hidden layers, the number of neurons per hidden layer, the activation function, and the training algorithm^39^. Sevenfold cross validation was used for training each ANN structure and validating it seven times each time on six different subsets and one subset^40^. The selected ANN structure is based on the hyperparameters that achieve the lowest average mean-square error on the seven validated subsets through the grid search cross-validation hyperparameter tuning process.

The hyperparameter search space was defined in two stages. In the first stage, the ANN architecture was tested using the following specifications: hidden layers ranging from 1 to 3 with neurons in each layer in the range of 5 to 25 (descending), activation functions of logsig, tansig, radbas, and radbasn, training algorithms of trainlm and trainbr), and L2 regularization coefficients from 0 to 10⁻^5^. In the second stage, using the best performing configuration found in the first stage as a base, an additional search was performed on deepening the network to 3–4 hidden layers with numbers of neurons ranging from 5 to 25 per layer (descending). During this step, logsig, tansig activation function were tested, with trainlm, trainbr, and trainrp as a learning algorithm at L2 regularization coefficients around 10⁻^5^. Network weights were initialized using the Nguyen-Widrow method, which scales the initial weights such that neurons are active in their sensitive fields and effectively covers the input domain at the beginning of training. L2 regularization was used as a way of decreasing overfitting which was tuned during the hyperparameter tuning in the range 10⁻^5^ to 0. Early stopping is enforced using a validation-based stopping criterion (max_fail = 30) such that training was stopped when the validation performance did not improve after 30 consecutive epochs^23^.

To ensure stable convergence, a minimum gradient threshold of 10⁻⁷ was also employed. Generalization was approached through sevenfold cross-validation generating multipoint measurements and assessment of consistency between training performance and validation performance within folds. The selected ANN architecture, which achieved the lowest average MSE on the seven folds, is then trained, validated and tested on the full 70% training data, with the remaining 15% validation and 15% test. Model accuracy is measured via the correlation coefficient (R) and mean-square error (MSE)^41^. While random splitting is widely adopted in similar studies, source-aware validation represents a more stringent protocol for multi-source datasets; this aspect is therefore acknowledged as a limitation of the present work.

### Optimization

The GA is a population-based metaheuristic optimization technique inspired by the principles of natural selection and evolution. It is widely recognized for its effectiveness in solving complex single- and multi-objective optimization problems across various domains, including applications that use AI with renewable energy systems^42^. The GA has demonstrated strong optimization performance when integrated with an ANN in SWT applications, as previously presented in Table 1, which encouraged its use with an ANN in this study. MATLAB R2023b was used throughout the optimization process, which begins with random initialization of a population, where each individual represents a unique combination of design parameters and operating conditions. The developed ANN models workers as a fitness function that receives each individual and predicts the corresponding ${C}_{p,ANN}$. The selection step uses elitism to preserve the best-performing individuals, while new offspring are generated via the Laplace crossover operator to explore the design space effectively. A power mutation operator is then applied to increase diversity and mitigate premature convergence by escaping local optima. The GA parameters presented in Table 3 are carefully calibrated to maintain a robust balance between exploration and exploitation, thereby ensuring efficient convergence toward the optimal solution.

**Table 3:** Parameters settings of genetic algorithm (GA).

Parameter	Value
Population size	450
Max. number of Generations	100
Elitism rate	0.05
Crossover rate	0.8
Mutation rate	0.15

### CFD modeling

High-fidelity transient computational fluid dynamics (CFD) simulations serve two critical functions within the optimization framework: (1) generating targeted, high-quality data points for iterative ANNs presents model enhancement, and (2) providing a final, physics-based validation of the optimal design identified by the GA. The incompressible flow around the Savonius rotor was modeled by solving the 3D RANS equations via the finite volume method in ANSYS Fluent.

The k-ω SST turbulence model was selected for its demonstrated accuracy in predicting flow separation and adverse pressure gradients prevalent in the complex, unsteady aerodynamics of Savonius turbines^12^. Rotor motion was explicitly captured via a sliding mesh technique to model transient rotor‒stator interactions. The pressure–velocity coupling was handled via the SIMPLE algorithm, whereas second-order upwind discretization schemes were applied to the momentum and turbulence transport equations for enhanced accuracy. The time step was selected to be equivalent to 1° of rotation as recommended by the following studies^8,12,13^. Convergence for each simulation was declared when the monitored torque and power coefficients varied by less than 1% and all equation residuals fell below 10⁻⁶ (with continuity below 10⁻^3^).

**Fig. 3:**
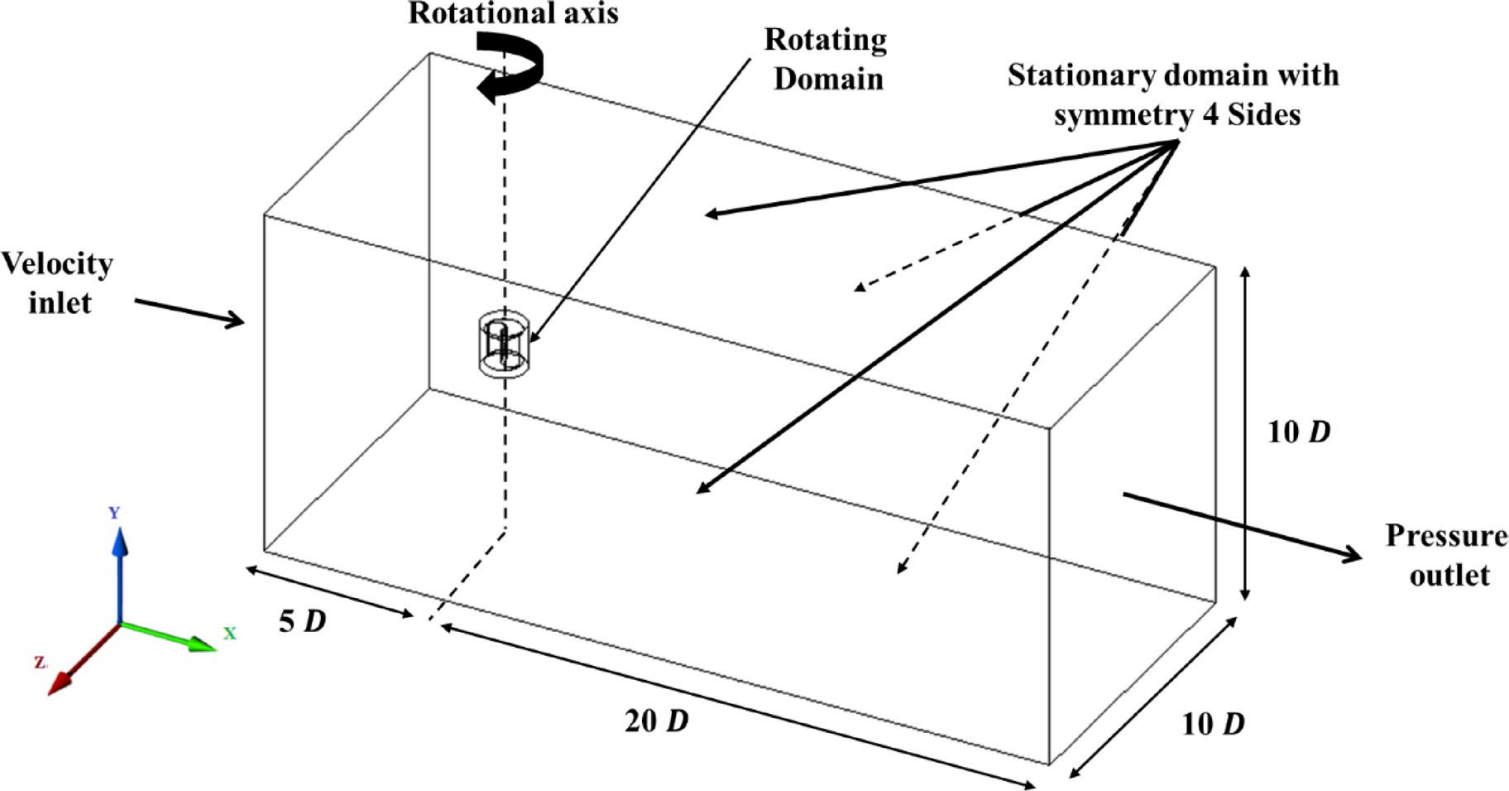
The 3D computational domain along with the boundary conditions.

**Fig. 4:**
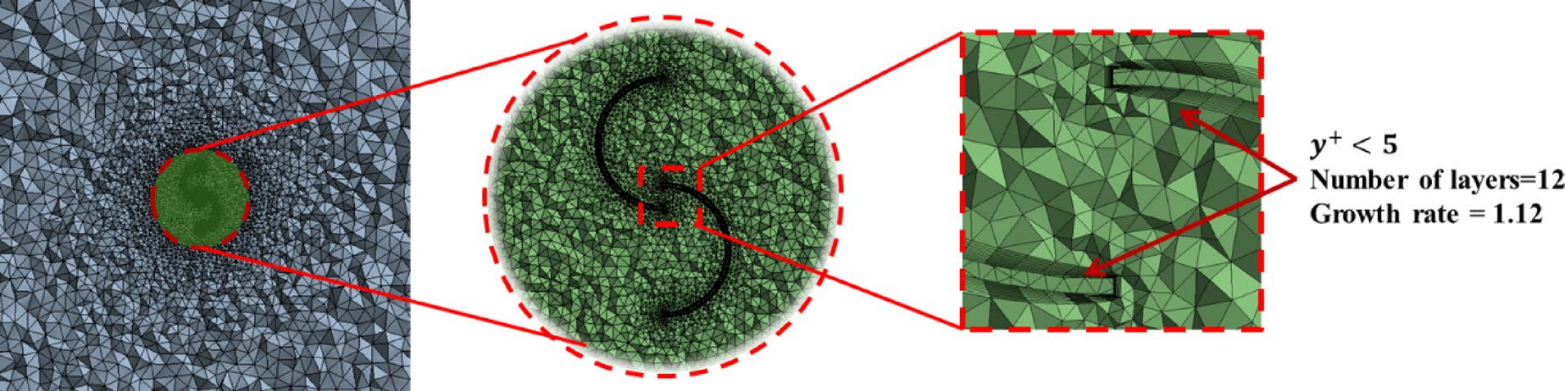
Computational mesh near the rotor and inflation layers over rotor blades.

The computational domain, illustrated in Fig. 3, was sized to minimize boundary interference, with the rotor center positioned 5D from the velocity inlet, 20D from the pressure outlet, and 5D from the lateral and top/bottom symmetry planes. A hybrid meshing strategy was employed (Fig. 4). The inner, rotating region containing the complex turbine geometry utilized an unstructured tetrahedral mesh, whereas the outer stationary domain employed a structured hexahedral mesh for computational efficiency. Attention was given to the near-wall resolution. A 12-layer inflation mesh with a growth rate of 1.12 was applied to all the blade surfaces, with the first cell height calculated to ensure a dimensionless wall distance of y⁺ < 5. This placed the first cell centroid within the viscous sublayer, ensuring appropriate application of the low-Reynolds-number k-ω SST formulation.

**Fig. 5:**
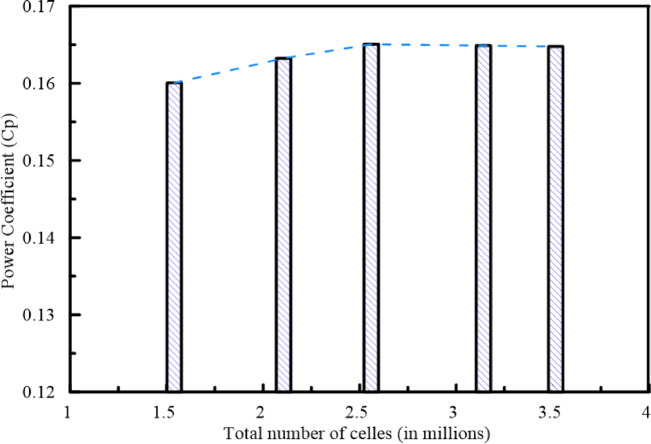
Power coefficient versus the number of cells at $\lambda$=0.8.

A systematic grid independence study was conducted using a straight Savonius rotor with the following geometry: AR = 1, OR = 0.15, EPR = 1.1, ${N}_{b}$=2 at a freestream velocity of 6 m/s and $\lambda$= 0.8. Five meshes ranging from 1.54 to 3.52 million cells were evaluated. As shown in Fig. 5, the solution converges from 2.56 million cells. Therefore, 2.56 million cells are used to reduce the computing time with a highly accurate solution. Despite the grid independent study was performed using straight rotor, the same meshing strategy and near-wall resolution criteria were consistently applied for all subsequent CFD simulated geometries.

### Sensitivity analysis

Monte Carlo-based sensitivity analysis was used with the developed ANN model to evaluate the influence of SWT design and operating parameters on performance. This method is suitable for nonlinear models such as ANNs, as it explores the input space and captures output variability. It has been used in different previous studies on engineering applications with ANNs^41,43^. The analysis is carried out in five steps. First, the input range for each parameter is defined as previously presented in Table 2. Second, each input parameter of SWT design and operating parameters is divided into 300 equally spaced steps across its range. At each step, the other parameters were randomly sampled to generate one million different combinations. Third, the developed ANN model is used to predict $\mathrm{C}\mathrm{p}$ for the million combination samples at each step, and the average $\mathrm{C}\mathrm{p}$ for each step is calculated. This ensures that the influence of the targeted parameter is isolated while accounting for interactions with other parameters. Fourth, the sensitivity index ${\Delta y}_{i}$, ($\Delta {y}_{i}=\mathrm{max}\overline{{C}_{p}}-\mathrm{m}\mathrm{i}\mathrm{n}\overline{{C}_{p}}$), is calculated for each input parameter, which is the difference between the maximum and minimum average $\mathrm{C}\mathrm{p}$ values across its 300 steps. Finally, the relative importance of each parameter is calculated by normalizing these indices. Mont Carlo sampling was performed using pseudo-random uniform distribution for continuous parameters and discrete sampling for categorical parameters. Convergence was verified by increasing both the Monte Carlo sample size and discretization resolution until negligible changes in the sensitivity indices were observed.

## Experimental setup

**Fig. 6:**
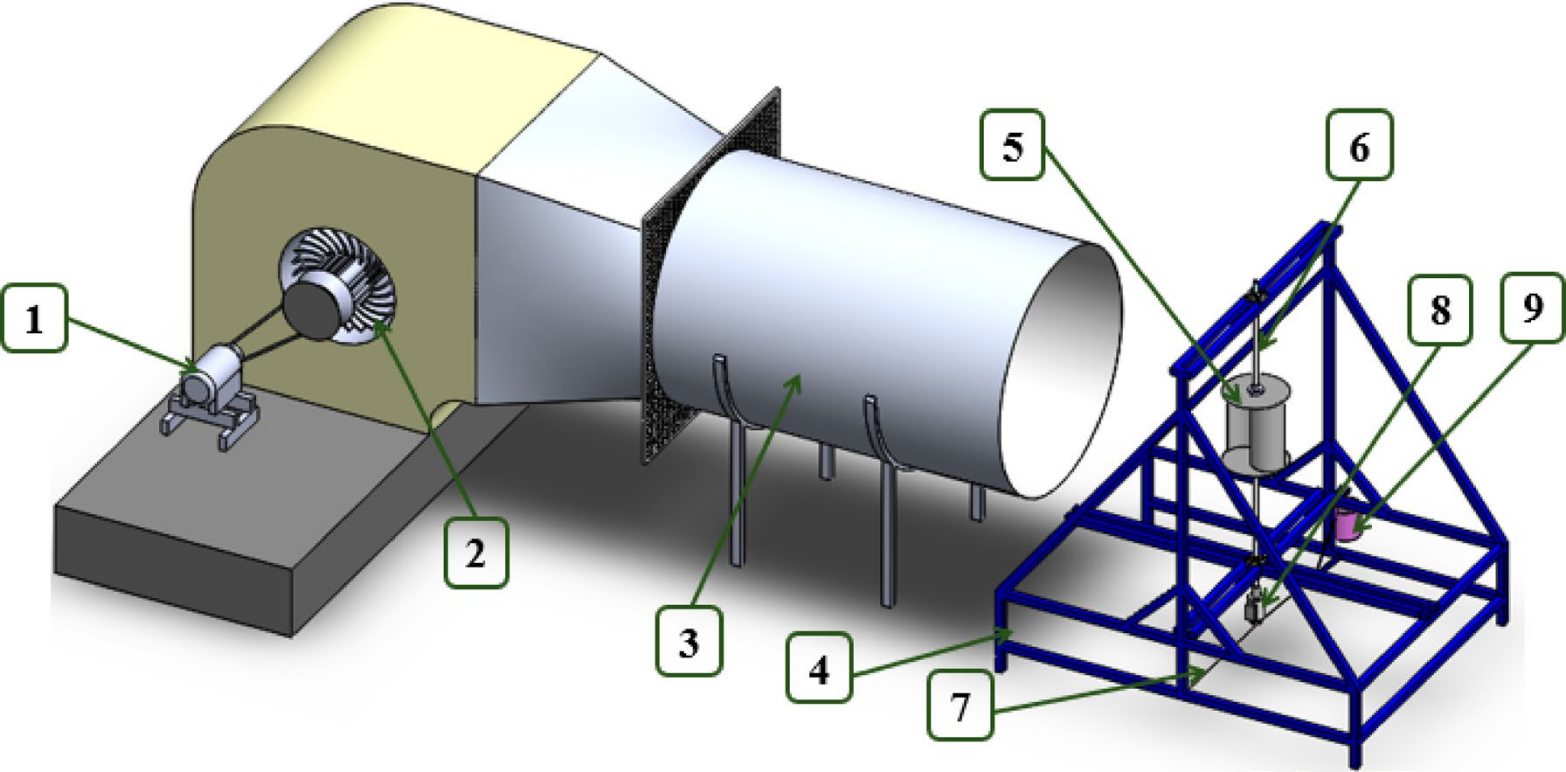
Test rig isometric view: (1) AC motor (2) Centrifugal fan (3) Circular duct (4) Steel frame (5) Rotor (6) Chrom shaft (7) Nylon String (8) Torque meter (9) Weighing pan.

Experimental tests were performed via a free-jet wind tunnel at the Advanced Fluid Mechanics Laboratory, Faculty of Engineering, Menoufia University, Egypt, to test the optimal SWT designs. This wind tunnel has been used previously for performance evaluation of vertical and horizontal axis wind turbines, as shown in Fig. 6^36,44,45^. The air flow through the wind tunnel is generated by a centrifugal fan driven by an AC motor. This AC motor rpm is precisely controlled via a variable frequency inverter, which controls the fan speed and, consequently, the wind speed. A grid of 9 measurement points was defined both vertically and horizontally to obtain the wind velocity distribution across the rotor projected frontal area (A). The local velocity (${V}_{i}$) at various points was measured via a pitot tube anemometer (Model: Extech HD350). The area-averaged wind velocity ($V$) incident on the turbine was calculated as:

7 $$V = \frac{1}{A}\smallint V_{i} { }dA$$

where $A$ is the projected area of the rotor and $\mathrm{d}\mathrm{A}$ is the area represented by each measurement point.

The tested rotor is mounted on a steel shaft connected to a digital shaft torquemeter (Model: MCRT 48801 V). The turbine was loaded mechanically via weights, which gradually increased the resistive torque and reduced the rpm until the breaking torque. For each load step, the system is allowed to reach a steady-state equilibrium at which the torque ($T$) and rotational speed ($N$) are recorded via a digital shaft torquemeter. The angular velocity (Ω) is then calculated from the rotational speed, and ${C}_{p}$ is determined via Eq. (6). The operating ranges and accuracies of the measuring devices are presented in Table 4.

**Table 4:** Specifications of the measuring instruments.

Instrument	Operating range	Accuracy
Pitot tube anemometer (Model: extech HD350)	0.4–30 m/s	± 0.03 m/s
Digital shaft torquemeter (Model: MCRT 48801 V)	0–11.3 N.m	± 0.011 N·m
	0–15,000 rpm	± 1 rpm

## Results and discussion

The integrated ANN-GA-CFD framework represented in methodology Section “Methodology” was applied to two cases:

- Case 1 (Straight-blade SWT): The design parameters for this case are the aspect ratio (AR), overlap ratio (OR), endplate ratio (EPR), and number of blades (${N}_{b}$) under varying operational conditions of the Reynolds number (Re) and tip speed ratio ($\lambda$).
- Case 2 (Twisted-blade SWT): The parameters considered in case 1 are the addition of the twist angle ($\emptyset$) as a key design variable.

### CFD model validation results

**Fig. 7:**
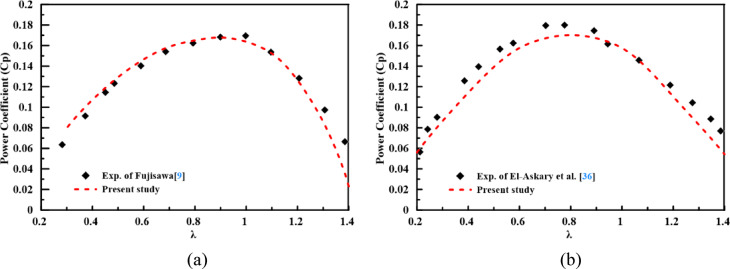
Comparison of CFD-predicted Cp with experimental data for validation cases: (**a**) Straight-blade rotor from Fujisawa^9^; (**b**) 45° twisted-blade rotor from El-Askary et al.^36^.

The CFD model presented in Section “CFD modeling” was validated on a straight rotor^9^ and a 45° twisted rotor^36^. For a straight rotor, the model shows good agreement with experimental tests along the operational $\lambda$ range, as presented in Fig. 7a. which is reflected in the coefficient of determination ${R}^{2}$=0.958 and MSE = $7.385\times {10}^{-5}$. For the twisted rotor, Fig. 7b shows that the simulated performance trend closely matches the experimental data, with ${R}^{2}$=0.931 and an MSE of $1.337\times {10}^{-4}$. The consistently high statistical metrics across both validation cases confirm that the employed CFD methodology, including the k-ω SST turbulence model, sliding mesh technique, and near-wall treatment, reliably captures the essential aerodynamics of semicircular Savonius rotors. This validated model forms a credible foundation for the subsequent data generation and design evaluation phases of this work.

### ANN-GA-CFD framework results

In the present work, the ANN-GA-CFD loop continues till convergence in the prediction of Cp between the ANN and CFD. The CFD data generated in each loop is fed to the training dataset used in the next loop, enabling progressive dataset enhancement. K-nearest neighbor-based density analysis was used to quantify data distribution before and after dataset augmentation. The data set sparsity index increases from 0.68 to 0.87, while the coverage ratio remains unchanged with a value of 0.5. This refers to more locally concentrated sampling distribution, which is consistent with the nature of the proposed ANN-GA-CFD framework. Unlike classical space filling techniques, the present approach employes a physical-guided sampling strategy as additional CFD samples are introduced in high performance insufficiently represented regions. Although the predefined parameter bounds remain unchanged, the explored data-supported region expands significantly, as evidence by the increase of convex hell volume from 402.93 to 723.52 (≈79.6%). This means that the additional CFD samples populate previously unvisited areas of parameter space. Moreover, the maximum interpermeate correlation decreases from 0.562 to 0.452, and the signal to noise ratio improved from 2.12 to 2.22 which indicates that the dataset quality was enhanced.

**Fig. 8:**
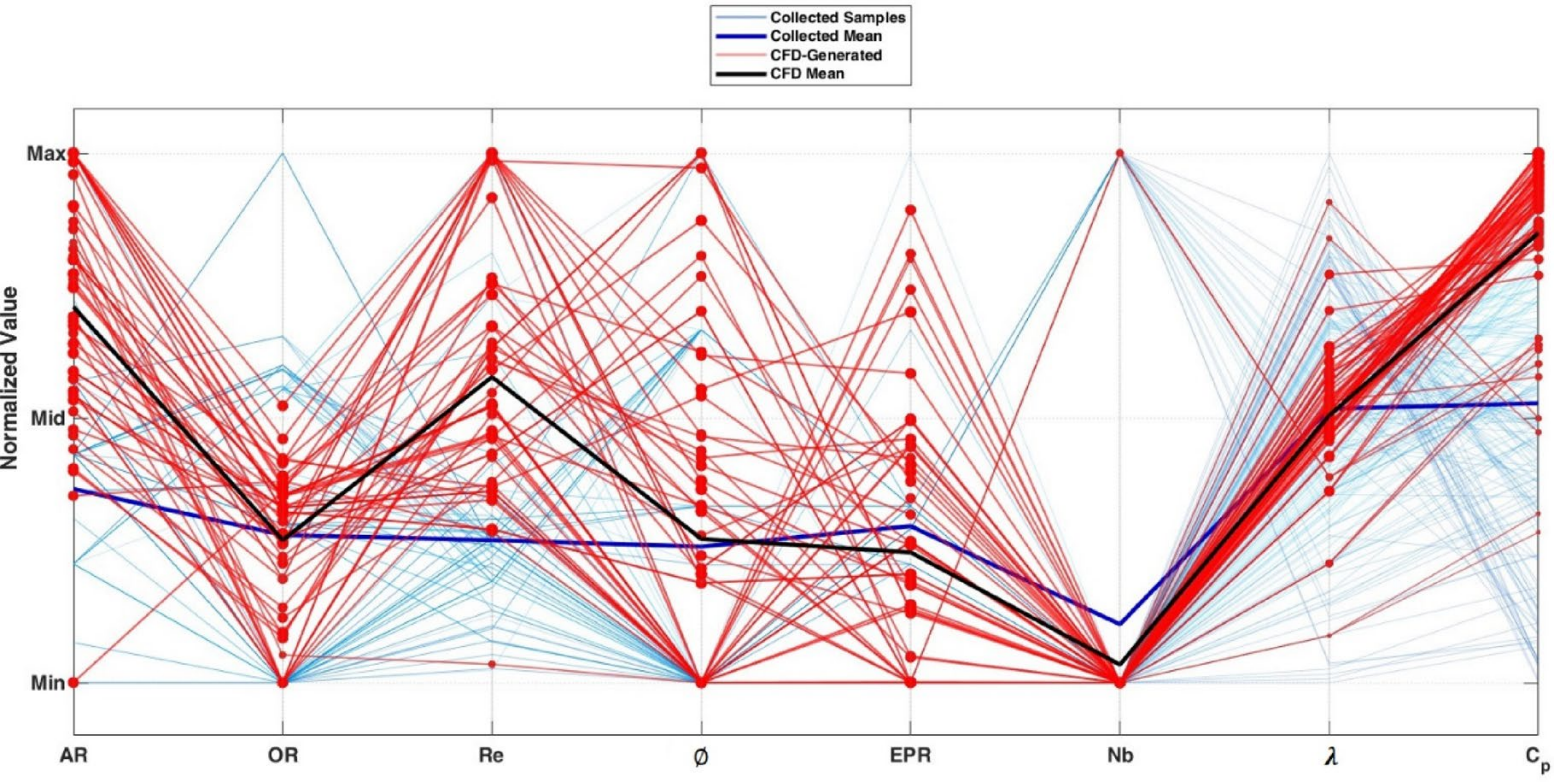
Parallel coordinates visualization of the design space, comparing collected data (N = 808, blue) with ANN-GA-CFD generated samples (N = 59, red gradient).

As illustrated in Fig. 8, the CFD-generated samples (red lines) complement the original dataset (blue lines) by targeting specific parameter combinations associated with higher Cp values. This shows distributed exploration rather than clustering around a narrow design space. Consequently, this targeted enrichment improves the representation of performance-relevant regions while maintaining coverage of the full predefined parameter ranges. The resulting dataset, which consists of 867 samples, is used to train the final ANN model for the twisted SWT. Whereas the subset corresponding to $\emptyset$ = 0°, which consists of 506 samples, is used for the straight SWT model.

The ANN-GA-CFD framework convergence was assessed based on $\left|\frac{{C}_{P,CFD}-{C}_{P,ANN}}{{C}_{P,CFD}}\right|\times 100<1\mathrm{\%}$. For the straight SWT configuration, convergence was achieved after 15 refinement cycles, supplemented by 11 additional CFD evaluations to verify physically consistent trends in Cp predictions across the parameter space. For the twisted SWT configuration, 33 refinement cycles were required due to increased design complexity, resulting in a total of 59 CFD simulations prior to convergence. This demonstrates the adaptive nature of the framework in addressing varying levels of nonlinearity and interaction among design parameters.

**Fig. 9:**
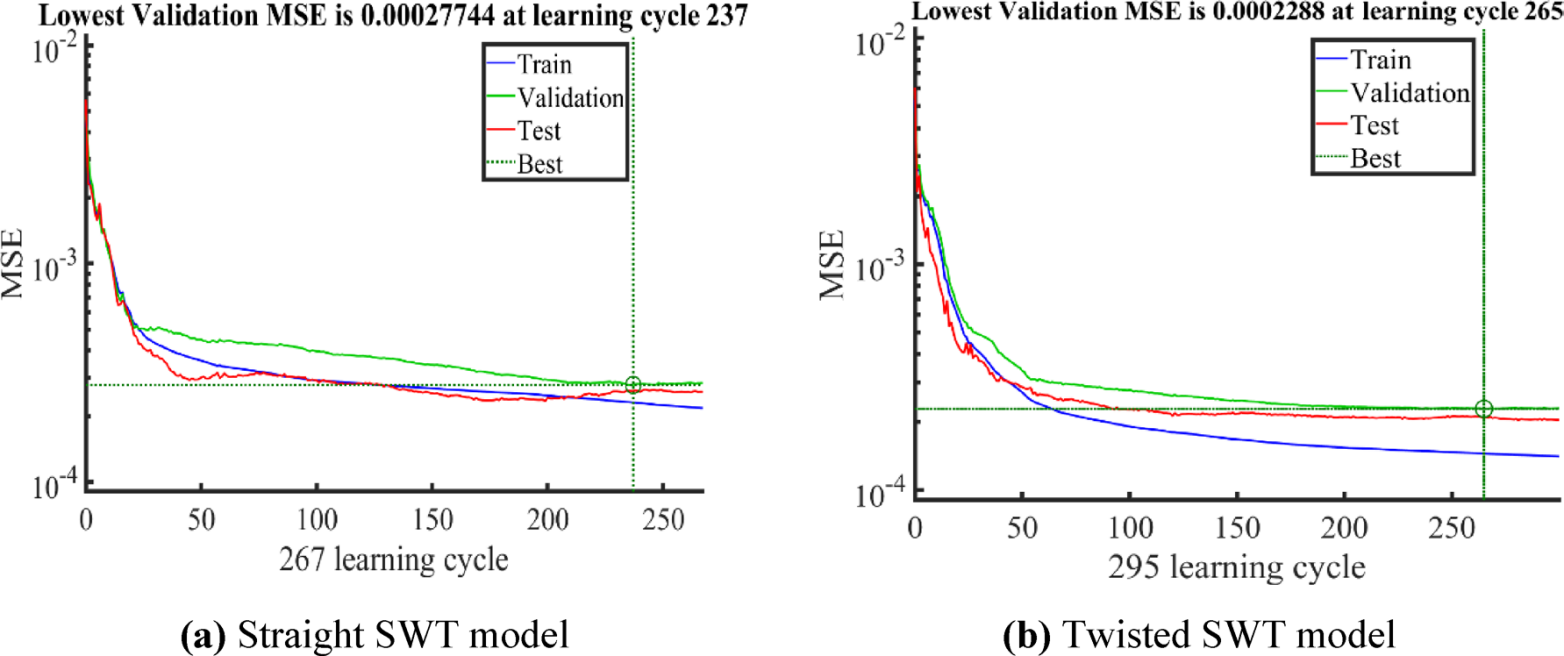
MSE variation during learning cycles of final models’ training, validation, and test for (**a**) straight SWT model and (**b**) Twisted SWT model.

The final ANN architecture, optimized via a grid search with sevenfold cross-validation hyperparameter tuning, yielded the lowest average MSE (~ 2 × 10⁻^4^) for the ANN structures represented in Table 5. The final trained model on the full 70% training data and validated and tested on the remaining 30% of the data yielded high R values of 0.965 and 0.98 on the training data for the straight and twisted SWT models, respectively, as represented in Table 5. Furthermore, Fig. 9 illustrates the performance curves of the final ANN models, showing the variation of MSE for training, validation and test datasets against learning cycles. The MSE decreases until reaching the minimum values of 2.77 × 10⁻^4^ and 2.28 × 10⁻^4^ on the validation data for straight and twisted SWT models respectively. The close agreement between training, validation and test curves without deviation reflects stable convergence and absence of overfitting in both models. This confirms the model ability to represent Cp in terms of the SWT design and operating parameters and serve as a fitness function with the GA to explore the SWT design space while searching for the optimal design.

**Table 5:** Specifications of the selected optimal ANN models.

SWT design	Hyperparameter tuning results				Lowest average	Final model performance		
	Neurons per layer	Activation fun	Training algorithm	Regularization	MSE on 7 folds	R-training	R-validation	R-Test
Straight SWT	(25,16,14,9)	Logistic sigmoid	Resilient backpropagation	0.00003	0.00017	0.965	0.9576	0.9612
Twisted SWT	(25,22,14,7)	Logistic sigmoid	Resilient backpropagation	0.0000135	0.0002	0.9806	0.9656	0.9627

**Fig. 10:**
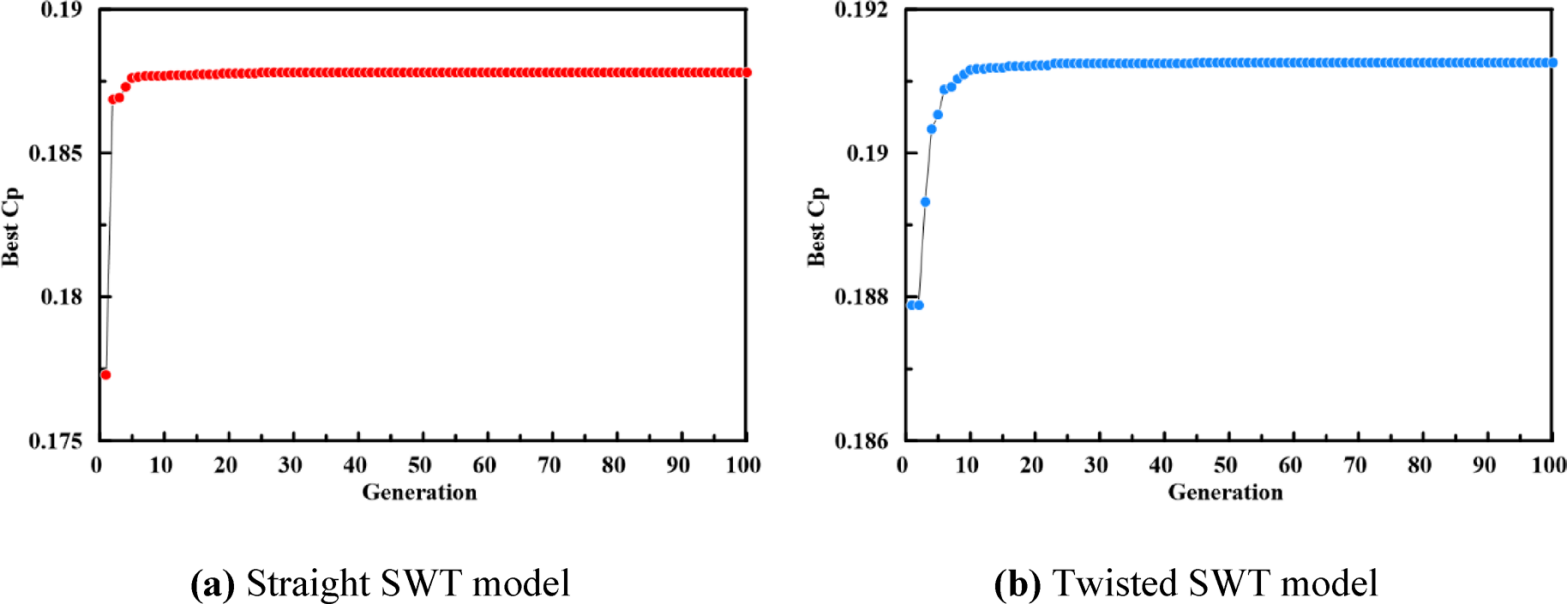
Best Cp versus number of generations during optimization for (**a**) Straight SWT model (**b**) Twisted SWT model.

Figure 10 illustrates the convergence history of GA, which exhibits a characteristic pattern: a sharp initial rise in the best–found ${C}_{p}$. This step is followed by a period of gradual refinement before the solution stabilizes at the global optimum. This convergence behavior validates the effectiveness of the selected GA hyperparameters in balancing exploration of the design space with local exploitation. The globally optimal designs for both straight and twisted Savonius rotors, as identified by the ANN-GA-CFD framework, are summarized in Table 6. The optimal straight and twisted configurations attained maximum power coefficient of 0.1856 and 0.1927 with performance improvement of 10.65% and 16.7%, respectively compared with the baseline design of Fujisawa^9^.

**Table 6:** Optimal design and performance results for straight and twisted SWTs.

Turbine	Parameters							Objective function		$\mathrm{D}\mathrm{e}\mathrm{v}\mathrm{i}\mathrm{a}\mathrm{t}\mathrm{i}\mathrm{o}\mathrm{n} \mathrm{\%}$
	AR	OR	Re	$\emptyset$	EPR	${N}_{b}$	$\lambda$	${C}_{P,ANN}$	${C}_{P,CFD}$	
Straight	1.197	0.1786	222,795.2	0	1.042	2	0.843	0.1878	0.1856	1.1853
Twisted	1.329	0.174	282,255.3	46.96	1.022	2	0.840	0.1912	0.1927	0.7833

**Fig. 11:**
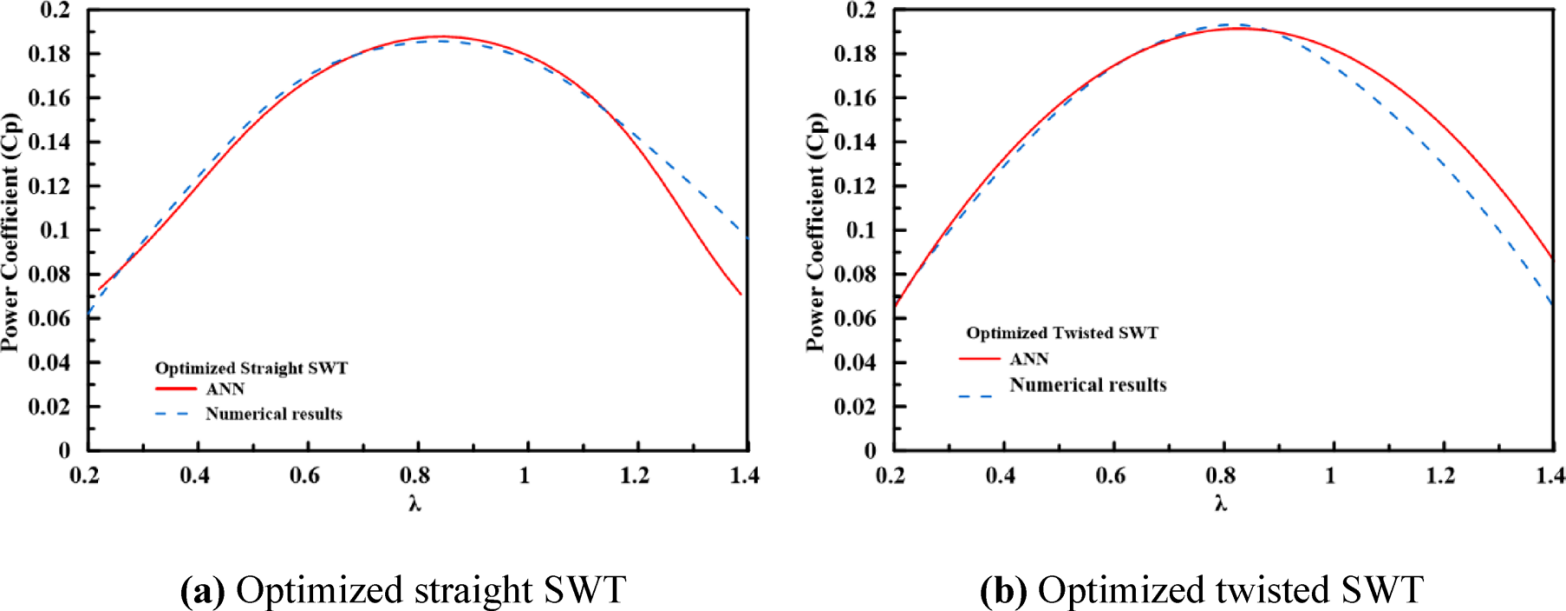
Comparison of ${C}_{p}$ versus $\lambda$ obtained from ANN and CFD for (**a**) the optimized straight-bladed and (**b**) the optimized twisted-bladed SWTs.

The predictive accuracy of the final ANN surrogate models was rigorously evaluated at the full operating range of $\lambda$ by comparing their performance predictions against high-fidelity CFD simulations for the optimized designs they identified. Figure 11 shows the ${C}_{p}$ versus $\lambda$ curves for the optimal straight and twisted rotors. The ANN and CFD predictions show good agreement. For both configurations, the ANN models accurately reproduce the complete performance characteristic, including the peak ${C}_{p}$ magnitude, the $\lambda$ at which it occurs, and the post-peak decline in efficiency. Quantitatively, the predictions exhibit a high ${R}^{2}>0.95$. Most critically, the deviation at the optimal operating point ($\lambda \approx 0.84$) is minimal, which is 1.185% for the straight blade and 0.78% for the twisted blade, as presented previously in Table 6. This close correspondence confirms that the ANN models trained on a hybrid multisource dataset and refined through the active learning loop have successfully internalized the essential aerodynamics of the Savonius rotor.

**Fig. 12:**
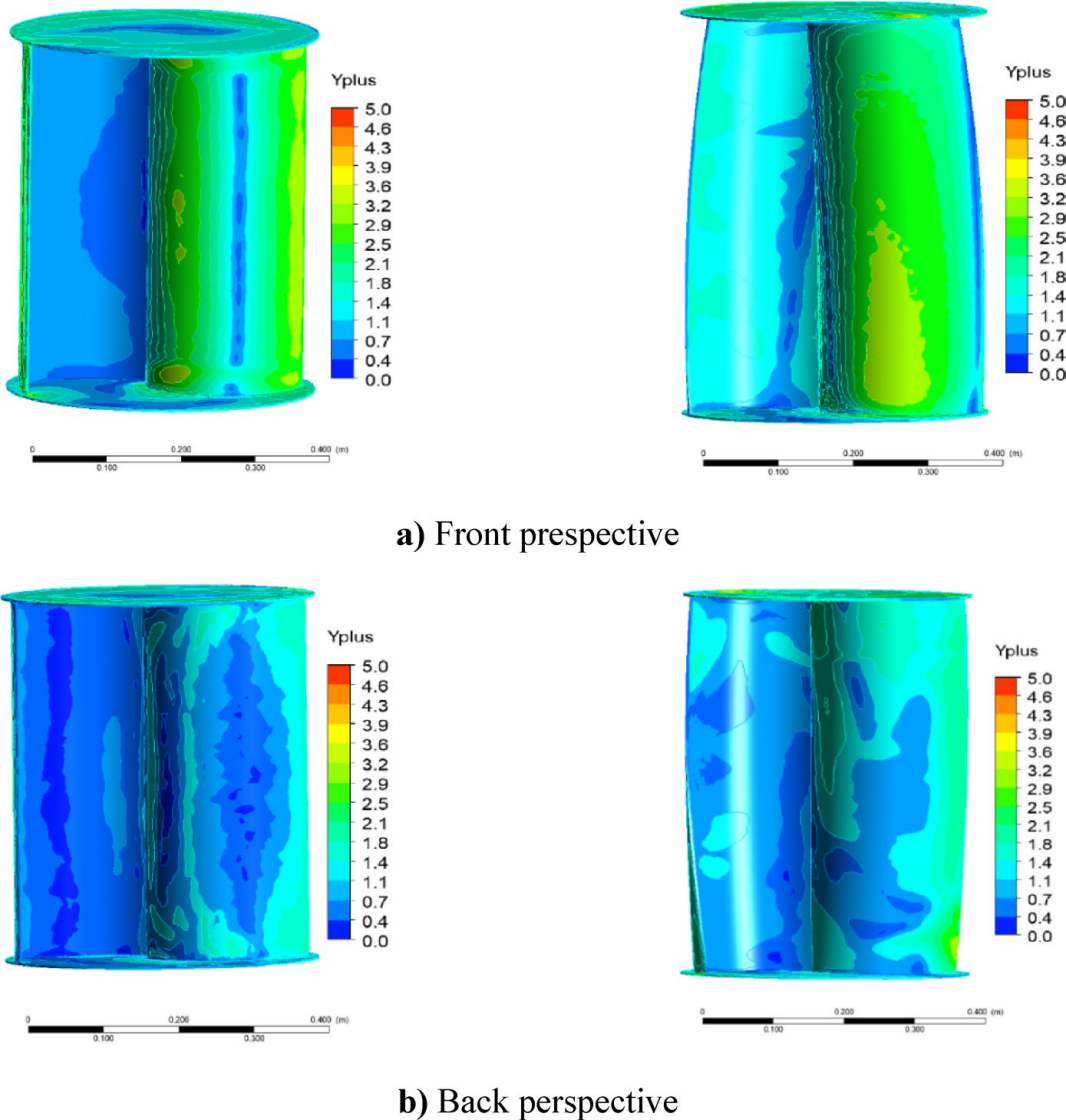
y⁺ distribution over the optimized straight and twisted rotors (a) Front perspective, (b) Back prespective.

The optimized twisted rotor employed approximately 3.1 million cells, while the optimized straight design utilizes approximately 3 million cells. In all CFD simulated cases the dimensionless wall distance y⁺ was kept below 5. Figure 12 presents y⁺ distribution over optimized straight and twisted geometries which confirms mesh accuracy preservation across all simulated configurations.

**Fig. 13:**
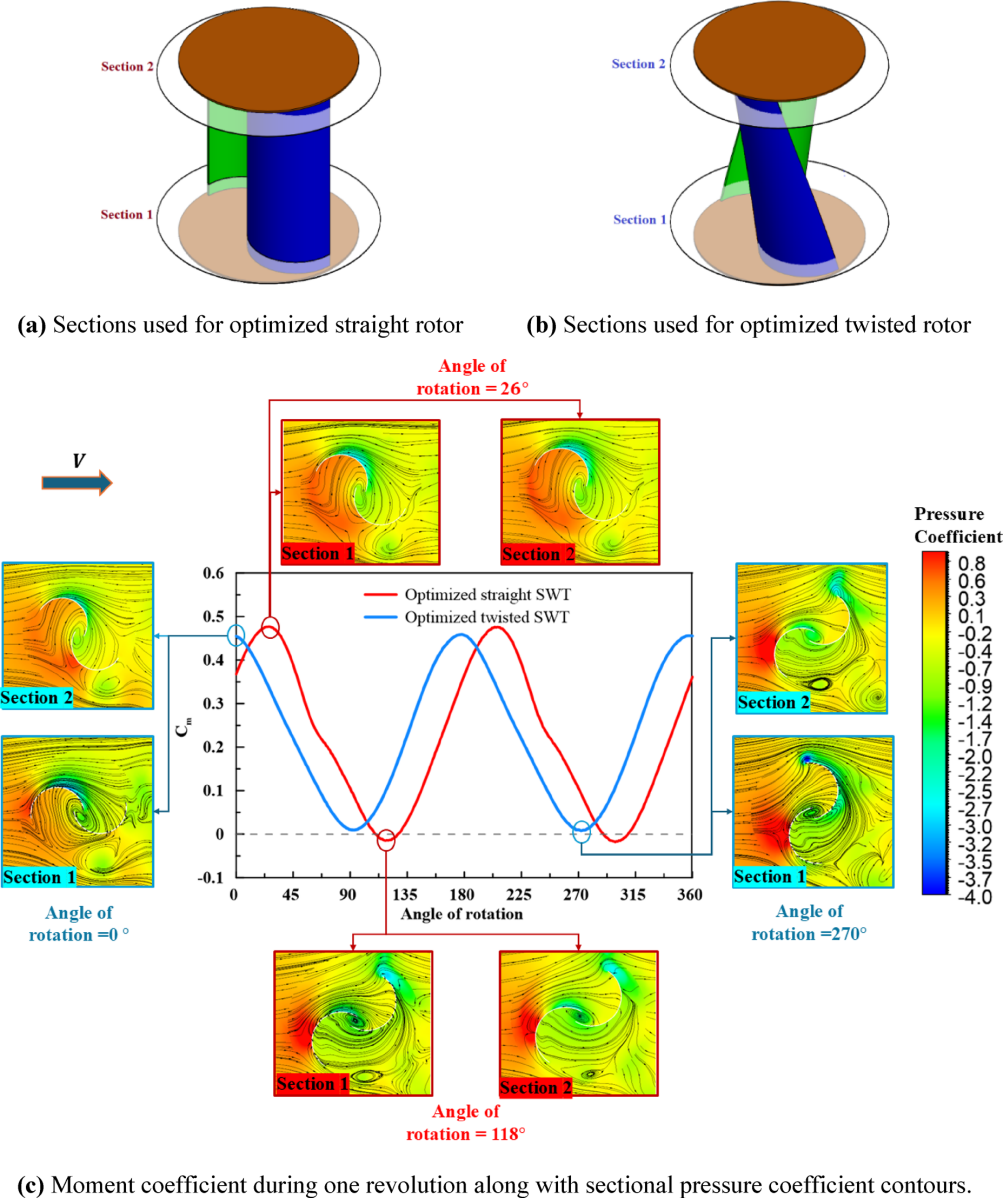
(**a**) Sections used for optimized straight rotor, (**b**) Sections used for optimized twisted rotor, and (**c**) Moment coefficient during one revolution along with sectional pressure coefficient contours for both optimized straight and twisted rotors.

The optimized rotors moment coefficient curves at the respective optimum operating conditions, see Table 6, are depicted in Fig. 13 through one revolution at optimum $\lambda$=0.84. The twisted rotor moment coefficient curve shows lower amplitude with higher average moment coefficient compared to the straight SWT rotor. Moreover, the twisted rotor moment coefficient indicates that the twisted optimized rotor at optimum operating conditions can perform positive moments for all angles of rotation. While the straight rotor shows negative moment at two regions through each rotational cycle (110$^\circ$–127$^\circ$) and (290$^\circ$–309$^\circ$). Figure 13 shows the pressure coefficient contours with streamlines for the optimized rotors at their critical angles of rotation. Two sections at 0.2 and 0.8 of rotor height for each rotor were selected to be presented as depicted in Fig. 13a, b aiming at showing the twist effect. The straight rotor blades orientation doesn’t change with height, while the twisted rotor blades orientation changes because of the twist effect.

In Fig. 13c, for straight rotor, Section 1 and 2 for each angle of rotation shows similar pressure coefficient contours with same orientations at both sections. At the peak moment that is achieved at 26 $^\circ$, The advancing blade high significant pressure at the concave region and low pressure at its convex surface results in high positive moment. While the lowest moment coefficient that appeared at 118 $^\circ$ results from the high-pressure zone on the returning blade convex side that leads to negative moment coefficient. For the twisted rotor, the rotor orientation and contours change from Section 1 to Section 2 at each angle of rotation due to the twist. The angle of rotation 0 $^\circ$ and 180 $^\circ$ causes peak moment coefficient while angle of rotation 90 $^\circ$ and 270 $^\circ$ leads to the lowest moment. The twist effect can be noticed from the interaction between blades and flow. At 0 $^\circ$ angle of rotation the advancing blade convex has high pressure zone near the tip at lower section (section 1) which can negatively affect the result moment, but the twist eliminates this zone at higher section (section 2). The same behavior is noted at 270 $^\circ$ where the twist reduces the high-pressure zone at the returning blade convex side and the vortices at the advancing blade tip for higher section compared to the lower section.

### Parametric analysis and performance trends

**Fig. 14:**
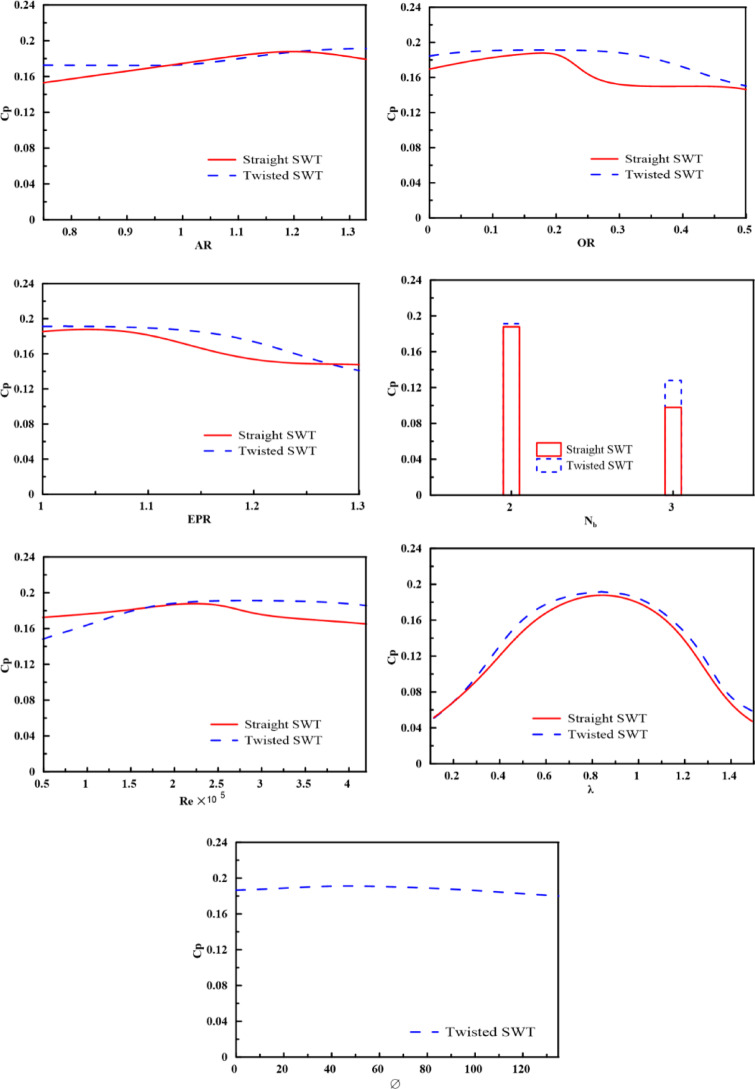
Parametric analysis of Cp for straight and twisted Savonius rotors, with each parameter varied independently while remaining parameters are fixed at their optimal values.

A comprehensive parametric study was conducted using validated ANN surrogate models to systematically evaluate the influence of key geometric and operational parameters on the power coefficient and to derive generalized design principles for Savonius wind turbines. First, as a one-dimensional parametric study, the developed ANN models were used to predict ${C}_{p}$ by varying each individual parameter while fixing the other parameters at their optimal values. Figure 14 shows the results. The red line represents the straight SWT, and the blue line represents the twisted SWT trend. ${C}_{p}$ increases with OR, EPR, Re, and $\emptyset$ until its optimal value then decreases. Interestingly, the twisted SWT performance increases with increasing AR even more than the maximum considered value through this study, reflecting its ability to manage adverse pressure gradients and maintain higher performance over a wider AR range. However, the twisted rotor shows slightly lower performance at low Re than the straight rotor does; the twisted rotor shows an ability to maintain higher performance over a wide range of high Re values than straight SWT, which shows an increase in performance until the optimal value is reached and then decreases. The ANN predictions and the GA optimization were limited to the parameter ranges explored, specified from the assembled dataset that previously presented in Table 2. Furthermore, the result for the reported optimum at AR = 1.329 was hence part of an interpolation on the limit of the investigated domain and not an extrapolation beyond it. Because the optimum lies at the upper bound, the results should not be interpreted as proof that the true global optimum could be found beyond the explored range, instead this means that exploring higher AR is a meaningful direction for future work.

While one-dimensional parametric studies provide insight into individual effects, the true aerodynamic response of Savonius turbines results from complex, multiparameter interactions. To elucidate these coupled relationships, two-dimensional performance contour plots were generated via the developed ANN models, which visualize the simultaneous variation in two key parameters while the other parameters were fixed at their optimum values. The ANN-generated performance contours in Fig. 14 confirm the global optima identified by the GA and show the nonlinearity of the relationship between ${C}_{p}$ and the SWT geometric and operating parameters.

**Fig. 15:**
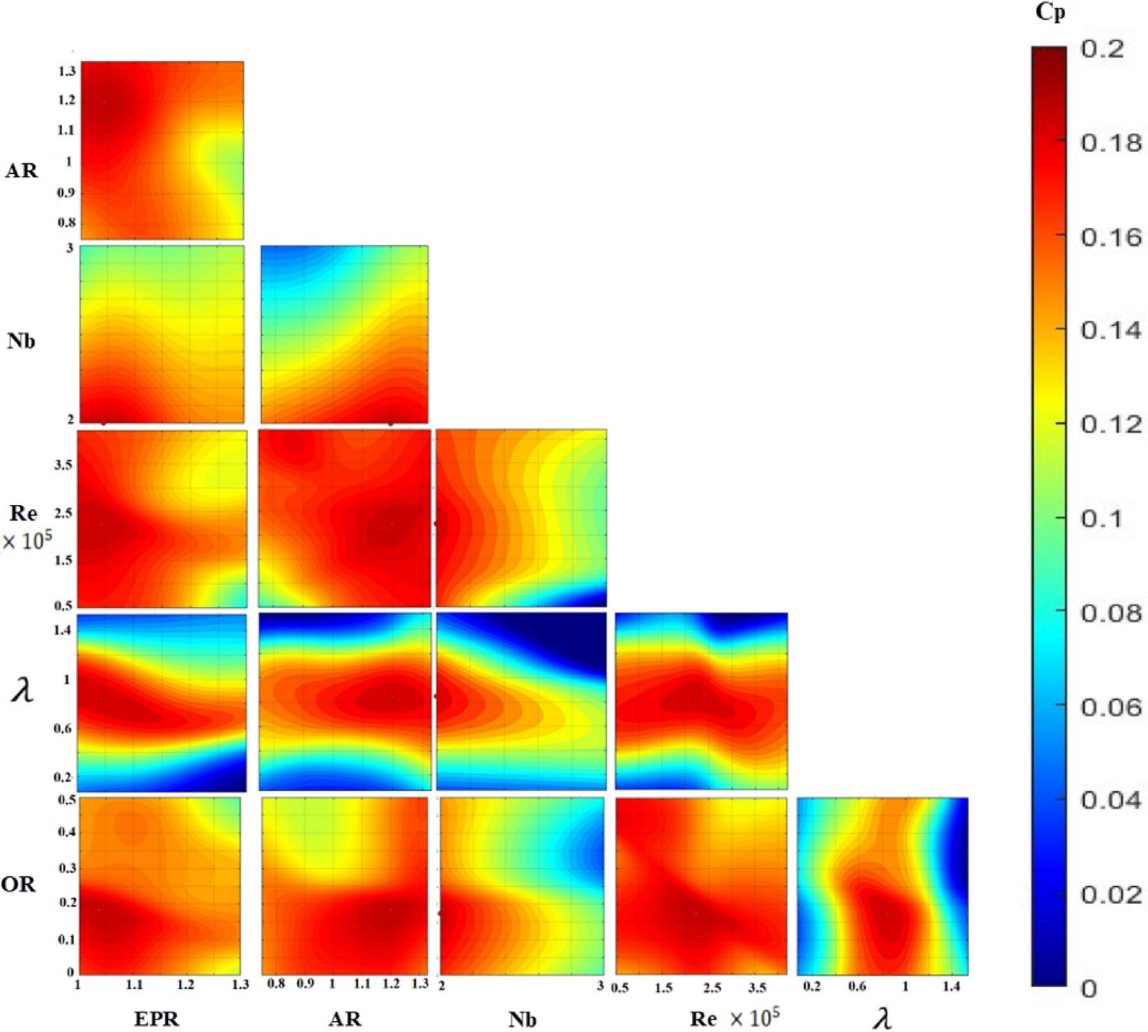
Contour plots of ${C}_{p}$ for straight-bladed SWT, showing the interaction between two parameters while remaining parameters are fixed at their optimal values.

**Fig. 16:**
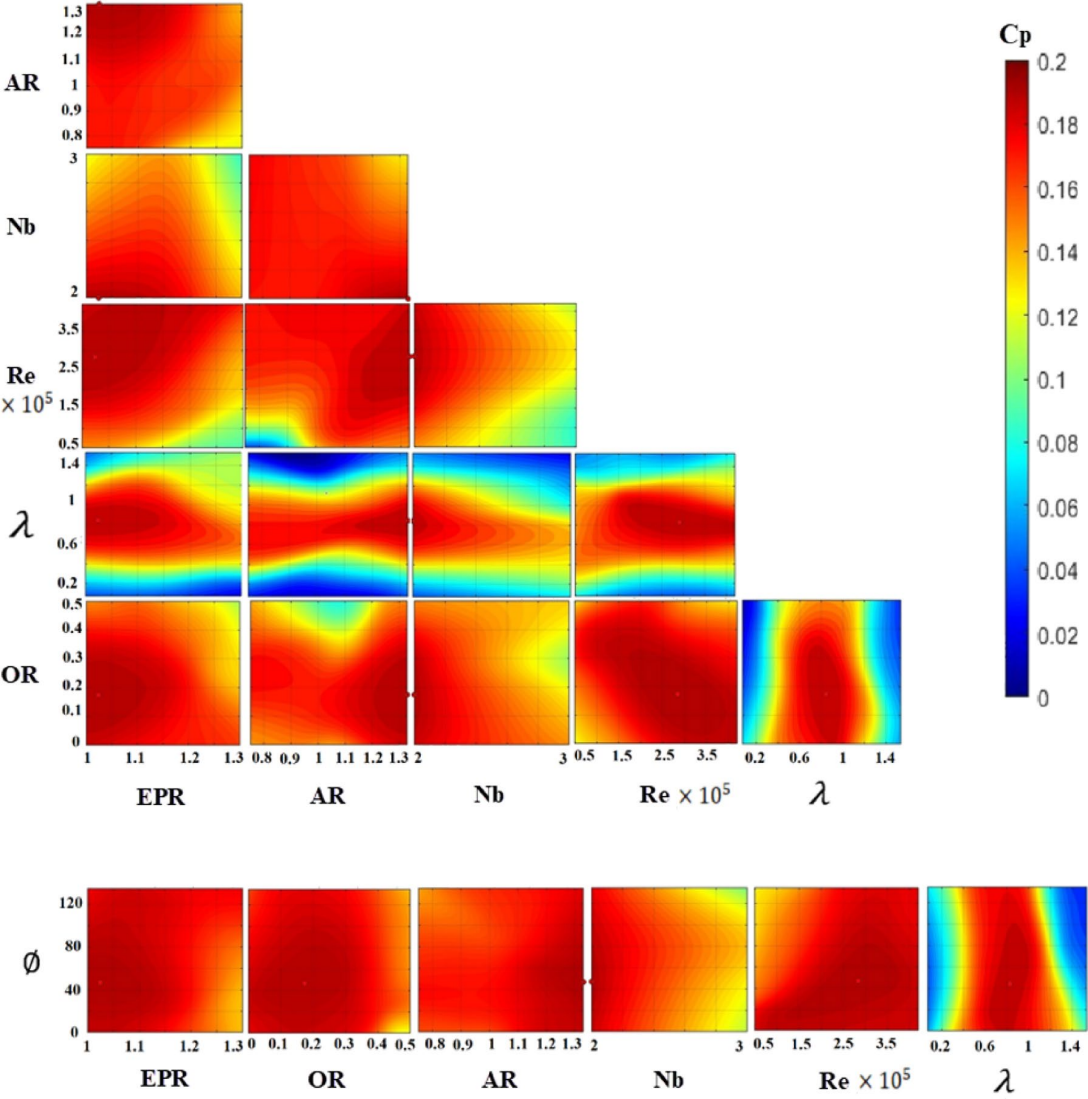
Contour plots of ${C}_{p}$ for twisted-bladed SWT, showing the interaction between two parameters while remaining parameters are fixed at their optimal values.

Figures 15 and 16 present contour plots of ${C}_{p}$ for straight and twisted-bladed SWT, respectively, showing the interaction between two parameters while the remaining parameters are fixed at their optimal values. A comparison of the contour plots for the optimal twisted and straight rotors confirms the ability of the optimal twisted rotor to maintain high performance at higher ARs over a wider range of Re values. This is represented as a larger red dark area at the (AR-Re) contours in Fig. 16 for the twisted rotor than for the straight rotor in Fig. 15. This trend is especially pronounced at higher Re values, which aligns with observations by Zhao et al.^7^ and Torres et al.^27^. However, their data were not included in this study because they were outside the considered parameter range; these findings reinforce the potential for performance gains with further AR increases, considering the suitable optimal twist angle. The high twist angle at a low AR shows poor performance, as represented by the yellow upper west corner of the AR-twist contour in Fig. 16, which agrees with the findings of Velásquez et al.^46^. Although the high twist shows low performance at high Re and low AR shows poor performance at low Re, see Re-Twist in Fig. 16.

### Sensitivity analysis results

**Fig. 17:**
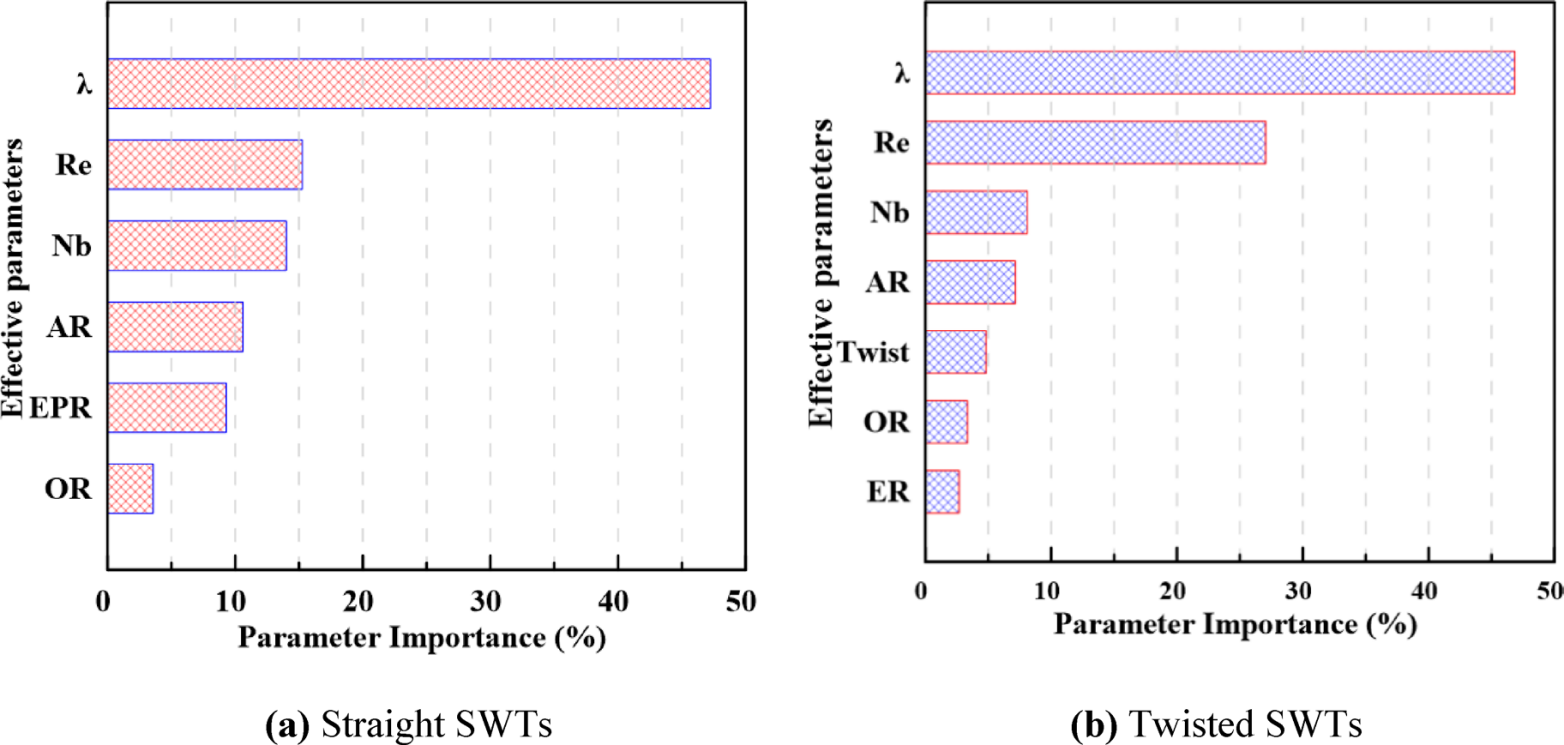
Global sensitivity analysis results showing the relative importance of input parameters on the power coefficient for (**a**) straight and (**b**) twisted Savonius wind turbine configurations.

Owing to the SWT multidimensional design space, one-dimensional or even two-dimensional parametric analyses are insufficient to capture the complex interactions among design and operating parameters. Therefore, a global sensitivity analysis based on the Monte Carlo method is employed, as discussed in Section “Sensitivity analysis”, to quantify the relative influence of each design and operating parameter on ${C}_{p}$. The sensitivity analysis results for both straight and twisted SWTs via the developed ANN models for each case at the parameter ranges in Table 2 are presented in Fig. 17. Compared with the geometric parameters, the operating parameters have a higher importance percentage. Compared with the other parameters, the tip speed ratio is the most effective parameter for SWT performance, with significantly high values for both straight and twisted SWTs. This means that adjusting turbine rpm with variations in the wind velocity and load is highly important for extracting the largest amount of energy from the available wind. Re, ${N}_{b}$ and the AR importance percentage follow $\lambda$ in descending order for both straight and twisted SWTs. The twist angle, OR and EPR have low influence percentages in descending order for twisted SWTs. However, straight SWTs have a greater importance percentage for EPR than for OR. A comparison of the effective parameter importance percentages for straight and twisted SWTs reveals that, compared with the other parameters, the SWT twist has a lower influence percentage, and the twist variation increases the influence of Re and reduces the influence of the SWT design parameters. To highlight the computational advantage of the ANN surrogate over high-fidelity CFD simulations, the cost of the sensitivity analysis was quantified. Approximately 1.8 × 10^9^ ANN evaluations were performed, requiring only $2.45\times {10}^{3}$ s (≈ 41 min) on a standard laptop (Intel Core i7-9750H CPU, 2.60 GHz, 32 GB RAM), demonstrating the efficiency of the proposed approach for large-scale parametric studies.

### Experimental testing of the optimum designs

**Fig. 18:**
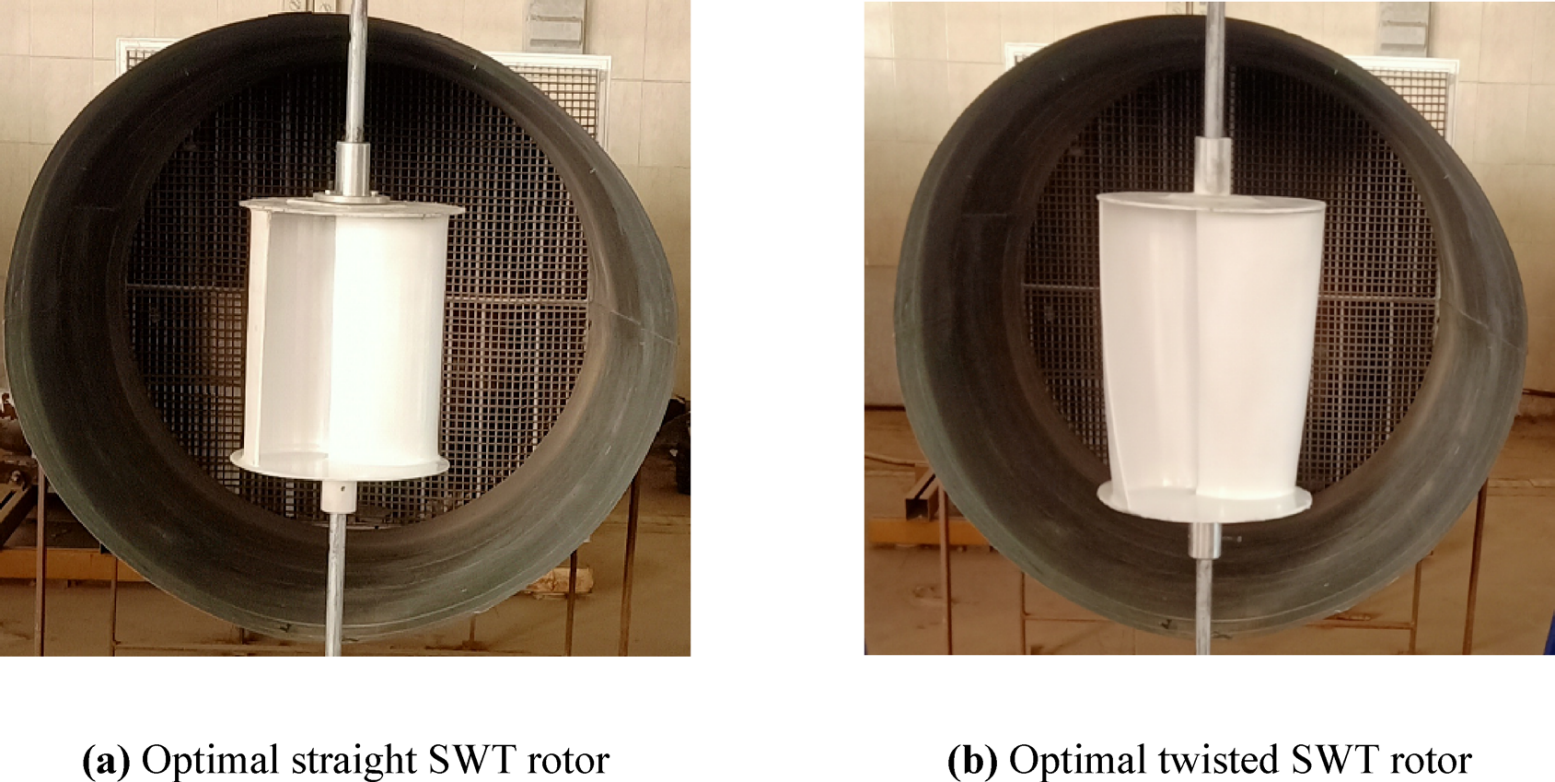
Manufactured optimal designs of (**a**) Straight SWT rotor and (**b**) Twisted SWT rotor.

To conclusively validate the data-driven optimization framework, the optimal straight and twisted blade designs are manufactured via 3D printing and tested, as shown in Fig. 18. The optimal straight and twisted prototypes were manufactured using fused deposition modeling (FDM) 3D printing technique with PLA filament at a nominal layer height of 0.20 mm. After fabrication, the 3D printed parts were subjected to post-processing consisting of support removal and light surface sanding on printed surfaces to reduce local printing irregularities, but without altering the designed geometry. Regarding the blade-surface finish, no separate profilometry measurement was performed; however, the residual surface texture was that typically associated with FDM-printed polymer components. After fabrication, the main dimensions of the straight and twisted prototypes, including rotor diameter, rotor height, end-plate diameter, and overlap distance were measured with a digital caliper and compared to the CAD geometry. The observed deviations were about ± 0.5 mm, which is compatible with the expected tolerance of FDM-printed PLA components and is small compared to the rotor scale utilized in the current experiments.

In the CFD model, the blade surfaces were therefore considered as smooth no-slip walls, and the near-wall region was resolved using 12 inflation layers with ${y}^{+}<5$. Accordingly, no equivalent sand-grain roughness was prescribed in the wall-boundary treatment. It is worth mentioning that neglecting the small residual printed-surface roughness is a modeling simplification that may contribute to the minor difference between numerical and experimental results.

**Fig. 19:**
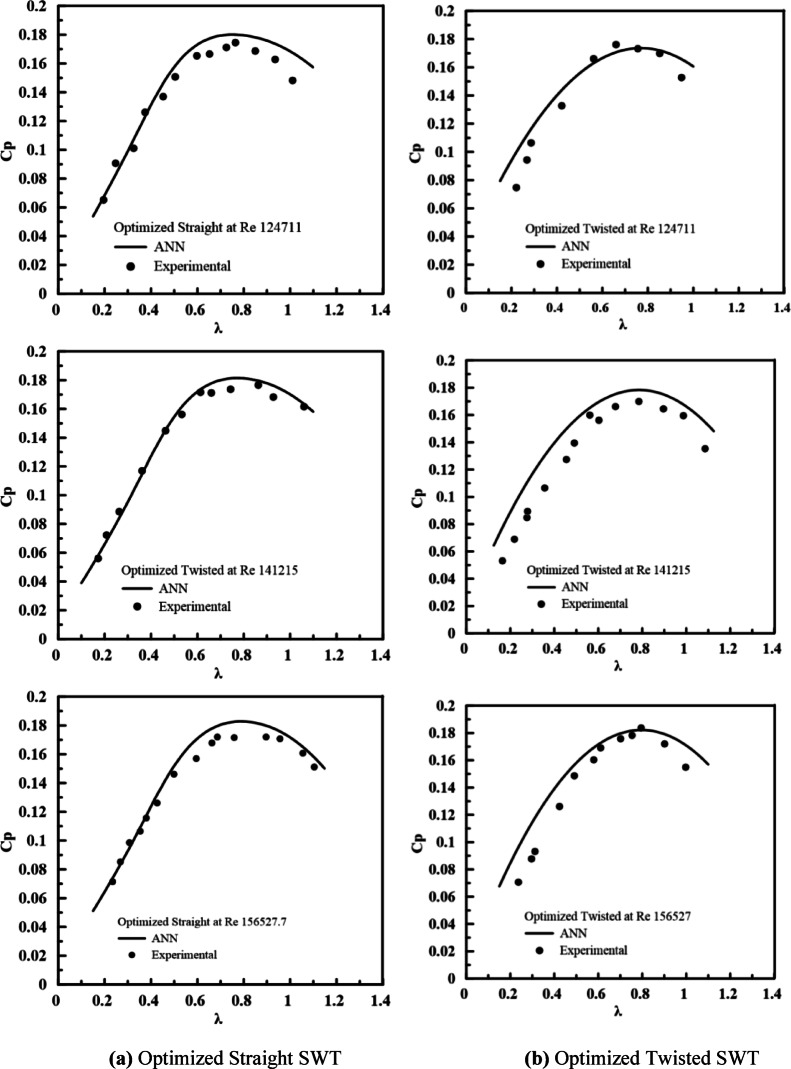
Comparison of experimental measurements and ANN prediction for optimized (**a**) straight and (**b**) twisted SWTs performance at different Re.

The performance was evaluated under three different operating conditions (Re ≈ 1.25 × 10^5^, 1.41 × 10^5^, and 1.57 × 10^5^) to assess the predictive accuracy across a practical operational range. The rationale for selecting the three Reynolds numbers was because the wind speed of the wind tunnel was limited to 10 m/s. Therefore, the experimental tests were performed at three representative Reynolds numbers that could be achieved reliably within the operating capability of the test facility. The experimental power coefficient measurements are compared with the ANN predictions in Fig. 19. Experimental results show a clear Reynolds number dependence, where the twisted rotor underperforms at low Re (− 0.85% at Re ≈ 1.25 × 10^5^) but outperforms the straight rotor at higher Re (up to 4.09%), consistent with ANN predictions indicating enhanced twisted rotor performance with increasing Reynolds number.

The measurement uncertainties, evaluated via the Kline-McClintock method^47^, are summarized in Table 7; the uncertainty in ${C}_{p}$ was ± 1.875%, ensuring confidence in the experimental results. Good agreement is observed for both configurations across all the tested λ and Reynolds numbers. The ANN models accurately predict the characteristic performance curves, including the rise to peak ${C}_{p}$ and the subsequent decline, with R^2^ reaching 0.956. All the experimental data points lie within the prediction uncertainty bounds, confirming the surrogate model’s reliability. The close correspondence between the experimental measurements of the optimal SWT designs and the ANN model predictions validates the accuracy of the proposed ANN-GA-CFD framework. This agreement highlights the framework’s effectiveness in assimilating multisource preprocessed datasets to achieve reliable design optimization for SWTs. Furthermore, the trained ANN model offers a scalable predictive platform capable of evaluating turbine performance over design and operating parameter space. When supplemented with turbine-specific operating data, the model’s predictive fidelity can be significantly enhanced, enabling it to function as an adaptive, control-oriented model that accurately represents turbine behavior across varying operating conditions.

**Table 7:** Measurement uncertainty of the experimentally measured parameters.

Parameter	Uncertainty
$\lambda$	± 0.455%
C_p_	± 1.875

## Conclusion

In this work, an integrated ANN-GA-CFD framework was developed for the optimization of semicircular Savonius wind turbines and the prediction of their performance on the basis of a comprehensive multisource dataset augmented by targeted CFD simulations. The proposed approach enables systematic investigation of not only geometric design but also operating parameters while maintaining computational efficiency through the use of ANN surrogate models. The implementation of the proposed framework resulted in two optimized SWT designs with straight and twisted blades. The optimized straight rotor ($AR$ = 1.197, $OR$ = 0.178, $EPR$ = 1.042, and ${N}_{b}$ = 2) achieved a maximum power coefficient (${C}_{p,max}$) of 0.1856. Furthermore, the optimized twisted rotor ($\emptyset$ = 47°, $AR$ = 1.33, $OR$ = 0.174, $EPR$ = 1.022, and ${N}_{b}$= 2) exhibit higher ${C}_{p,max}$ of 0.1927. The optimal straight and twisted configurations achieve performance improvement of 10.65% and 16.7%, respectively compared with the baseline design of Fujisawa^9^ which achieved a ${C}_{p,max}$ of 0.169.

The Monte Carlo-based global sensitivity analysis identified the operating parameters, particularly the tip speed ratio and Reynolds number, as the most important factors affecting SWT performance, followed by the main geometric parameters, including ${N}_{b}$, $AR$, $\emptyset$, $OR$, and $EPR$, in descending order of influence. The dominant effect of the tip speed ratio highlights the importance of appropriate rotational speed control under varying wind conditions. The sensitivity of SWT performance to operating conditions, particularly $Re$, varies with the twist angle ($\emptyset$), and a suitably chosen twist can result in more stable performance over a wide range of $Re$.

The experimental validation of the optimized SWT designs demonstrated good agreement with the ANN predictions, with a coefficient of determination (*R*^2^) of 0.956, confirming the accuracy and reliability of the developed surrogate models within the studied parameter ranges. The current validated ANN-GA-CFD framework provides a flexible predictive tool for SWT performance assessment and optimization. Future work will focus on extending the proposed ANN-GA-CFD framework to hybrid vertical-axis wind turbine configurations and exploring its integration into performance optimization and control strategies under different operating conditions.

## Data Availability

The data that supports the findings of this study are available from the corresponding author, [Ahmed. S. Saad], upon reasonable request.

## List of symbols

A: Projected frontal area [m^2^]
AR: Aspect ratio (H/D) [-]
${C}_{f}$: Local skin friction coefficient [-]
$${C}_{p}$$: Power coefficient [-]
${C}_{p,ANN}$: Power coefficient predicted using ANN [-]
${C}_{p,CFD}$: Power coefficient predicted using CFD [-]
${C}_{p,max}$: Maximum power coefficient [-]
$${C}_{t}$$: Torque coefficient [-]
d: Bucket diameter [m]
D: Turbine diameter [m]
${D}_{o}$: End plate diameter [m]
e: Overlap distance [m]
EPR: End plate ratio (D/${D}_{o}$) [-]
H: Rotor height [m]
MSE: Mean square error [-]
${N}_{b}$: Number of blades [-]
OR: Overlap ratio (e/d) [-]
${P}_{avilable}$: Available wind power [W]
R: Correlation coefficient [-]
${R}^{2}$: Coefficient of determination [-]
Re: Reynolds number [-]
${r}_{i}$: Normalized sensitivity index
T: Dynamic torque [N·m]
${u}_{t}$: Turbine’s tip speed [m/s]
$V$: Area-averaged wind velocity [m/s]
$${v}_{i}$$: Local velocity
$\overline{x }$: Parameter mean value [-]
$${x}_{i}$$: Parameter value
$${y}_{i. actu}$$: Actual value [-]
$\widehat{{y}_{i,act}}$: Mean actual value [-]
$${y}_{i. pred}$$: Predicted value [-]
$\widehat{{y}_{i,\mathrm{p}\mathrm{r}\mathrm{e}\mathrm{d}}}$: Mean predicted value

## Greek symbols

$\lambda$: Tip speed ratio [-]
$\mu$: Dynamic viscosity [Pa·s]
$\rho$: Air density [kg/m^3^]
$$\sigma$$: Standard deviation [-]
${\tau}_{w}$: Wall shear stress [Pa]
$\emptyset$: Rotor twist angle [°]
$\theta$: Rotor azimuth angle [°]
Ω: Angular velocity of the rotor [rad/s]
$${\Delta y}_{i}$$: Sensitivity index

## Abbreviations

AI: Artificial intelligence
ANN: Artificial neural network
CFD: Computational fluid dynamics
GA: Genetic algorithm
RANS: Reynolds-averaged navier–stokes
SIMPLE: Semi-implicit method for pressure-linked equations
SST: Shear stress transport
SWT: Savonius wind turbine
VAWT: Vertical axis wind turbine
